# Spliceosomal mutation drives melanoma tumorigenesis via lineage-specific RAS activation

**DOI:** 10.1126/sciadv.adz8289

**Published:** 2026-05-01

**Authors:** Ruixin Jiang, Peiqi Xing, Jindou Xie, Gang Bai, Yuzhu Zhang, Pengcong Hou, Hao Luo, Yanni Ma, Ruixin Liu, Yang Zheng, Xiangyu Chen, Bin Jiang, Jing Huang, Yanjie Zhang, A. Hunter Shain, Ming Lei, Robert L. Judson-Torres, Jing Ye, Zhaoqi Liu, Hanlin Zeng

**Affiliations:** ^1^ Department of Oncology, Shanghai Ninth People’s Hospital, Shanghai Jiao Tong University School of Medicine, Shanghai, China.; ^2^ Shanghai Institute of Precision Medicine, Shanghai Ninth People’s Hospital, Shanghai Jiao Tong University School of Medicine, Shanghai, China.; ^3^ Department of Computational Biology, China National Center for Bioinformation, Beijing, China.; ^4^ Beijing Institute of Genomics, Chinese Academy of Sciences, Beijing, China.; ^5^ University of Chinese Academy of Sciences, Beijing, China.; ^6^ Department of Oral and Maxillofacial-Head Neck Oncology, Shanghai Ninth People’s Hospital, Shanghai Jiao Tong University School of Medicine, College of Stomatology, Shanghai Jiao Tong University, Shanghai, China.; ^7^ Department of Plastic and Reconstructive Surgery, Shanghai Ninth People’s Hospital, Shanghai Jiao Tong University School of Medicine, Shanghai, China.; ^8^ Department of Dermatology, University of California, San Francisco, San Francisco, CA, USA.; ^9^ Helen Diller Family Comprehensive Cancer Center, University of California, San Francisco, San Francisco, CA, USA.; ^10^ State Key Laboratory of Oncogenes and Related Genes, Shanghai Jiao Tong University School of Medicine, Shanghai, China.; ^11^ Huntsman Cancer Institute, University of Utah, Salt Lake City, UT, USA.; ^12^ Department of Oncological Sciences, School of Medicine, University of Utah, Salt Lake City, UT, USA.; ^13^ Department of Dermatology, School of Medicine, University of Utah, Salt Lake City, UT, USA.; ^14^ Geriatric Department, Geriatric Medical Center, Shanghai Ruijin Hospital, Shanghai Jiao Tong University School of Medicine, Shanghai, China.

## Abstract

Mutations in splicing factors are recurrent across human cancers and drive widespread RNA splicing dysregulation. Among these, *SF3B1* is the most frequently mutated, yet its hotspot mutations exhibit lineage specificity, with *SF3B1*^R625^ mutations predominantly found in melanoma and *SF3B1*^K700E^ in hematologic malignancies. However, the mechanistic basis for this cancer-type specificity remains unclear. Here, we demonstrate that *SF3B1*^R625H^ induces greater activation of alternative 3′ splice site than *SF3B1*^K700E^. Mechanistically, the polyadenine-enriched sequence surrounding cryptic branch point sites confers *SF3B1*^R625H^ selective advantage in aberrant splicing. This splicing bias leads to preferential missplicing of *NF1*, a RAS inhibitor, resulting in RAS hyperactivation and accelerated melanoma progression in mouse models. This study redefines the oncogenic paradigm of *SF3B1* mutations by demonstrating that distinct hotspot mutations exploit lineage-specific splicing vulnerabilities to drive tumorigenesis and establishes RAS activation as key mechanism underlying *SF3B1*^R625H^-driven melanoma, positioning RAS pathway as tractable therapeutic target in *SF3B1*-mutant melanoma.

## Teaser

Spliceosome mutation ignites lineage-specific RAS activation in melanoma.

## INTRODUCTION

In eukaryotic cells, gene expression requires processing pre-mRNA into mature mRNA through the splicing of introns and joining of exons within the nucleus (*1*). Cancer cells can “hijack” this splicing machinery to induce specific splicing abnormalities that drive tumor initiation and progression (*2*, *3*). Comprehensive genomic analyses of cancer have identified recurrent mutations of splicing factors in human cancers, providing the most direct evidence of dysregulated splicing functions in cancer (*4*). Mutations in the core RNA splicing factor *SF3B1* are the most common across cancer types and occur as “hotspot” heterozygous point mutations (*5*). Analysis of the effects of *SF3B1* hotspot mutations on RNA splicing has consistently identified that these mutations use aberrant branch point nucleotides, manifesting in increased use of cryptic 3′ splice site (3′ss) and resulting in alternative splicing events (*6*, *7*). The resulting aberrantly spliced transcripts frequently lead to nonsense-mediated decay (NMD) and thus down-regulation of mRNA expression of hundreds of target genes (*6*).

The frequently mutated *SF3B1* residues display apparent exclusivity on different cancer lineages (*8*). For instance, the K700 residue of *SF3B1* is predominantly mutated in hematologic malignancies, including myelodysplastic syndromes (MDSs) (*9*, *10*) and chronic lymphocytic leukemia (CLL) (*11*, *12*), whereas mutations at position R625 are primarily associated with melanoma, including uveal melanoma (UVM), mucosal melanoma (MM), and skin cutaneous melanoma (SKCM) (*13*–*15*). The reasons of this cancer specificity are unclear.

Recent functional investigations of aberrant splicing, driven by mutant *SF3B1*, in the context of MDS (*16*), CLL (*8*), MM, UVM (*17*), and breast cancer (*18*) have begun to elucidate specific aberrant splicing events required for the maintenance of *SF3B1*-mutant cancers. In addition, studies in the context of myeloid leukemias have identified that *SF3B1* mutations confer therapeutic vulnerabilities to further modulation of splicing (*19*, *20*), to poly(adenosine 5′-diphosphate–ribose) polymerase inhibition (*21*), as well as to specific metabolic perturbations (*22*, *23*). These mechanistic insights highlight the role of *SF3B1* mutations in driving tumorigenesis by disrupting the splicing of genes across diverse cellular pathways, such as *MAP3K7*, *BRD9*, *PPP2R5A*, and others (*8*, *17*, *24*, *25*). However, these studies predominantly focus on the K700E mutation. There is now a deficiency in research aimed at uncovering the oncogenic mechanisms underlying R625 mutation-associated cancers. The *SF3B1*^R625H^ mutation is frequently observed in melanoma, with mutation frequencies ranging from 14 to 29% in UVM and 12% in MM (*6*, *15*). It remains unclear why *SF3B1*^R625^ mutation, but not *SF3B1*^K700E^ mutation, preferentially occurs in melanoma.

To address above questions, we introduced *SF3B1*^K700E^ and *SF3B1*^R625H^ mutations into both normal human melanocyte (NHM) cells and K562 chronic myeloid leukemia cell line to investigate residue-specific splicing abnormalities by the two different *SF3B1* hotspot mutations. We found that *SF3B1*^R625H^ leads to overall greater cryptic alternative 3′ss switch than *SF3B1*^K700E^ due to a R625H-specific intronic sequence with enriched polyadenines (poly-A’s) around the cryptic branch point site (BPS). Notably, *NF1*, a key RAS inhibitor, was preferentially misspliced and inactivated by the *SF3B1*^R625H^ mutation, leading to RAS activation and accelerated cell growth in vitro, as well as enhanced melanoma growth in mouse models. Our findings reveal distinct splicing preferences associated with *SF3B1* mutations and highlight *NF1* missplicing and RAS activation as critical drivers of *SF3B1*^R625^ mutation-mediated melanoma.

## RESULTS

### *SF3B1*^R625^ mutation leads to distinct missplicing profile compared to the *SF3B1*^K700E^ mutation

**Fig. 1.**
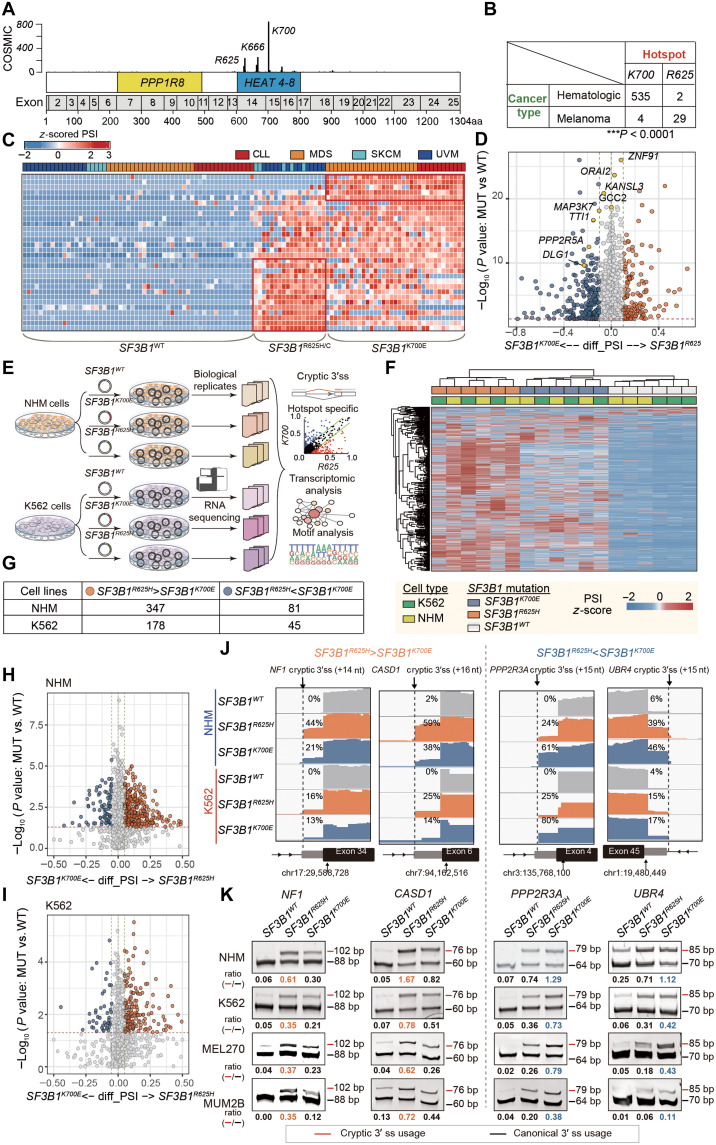
*SF3B1*^R625^ mutation leads to distinct missplicing profile compared to the *SF3B1*^K700^ mutation. (**A**) *SF3B1* mutation maps based on information from COSMIC database. The *x* axis represents exon numbers and amino acid positions. The *y* axis indicates the count of identified mutations. (**B**) Contingency table shows the number of patients with hematologic and melanoma cancers carrying different *SF3B1* mutations (Fisher’s exact tests, ***P < 0.001). (**C**) Hierarchical clustering and heatmap analysis of the top 30 cryptic 3′ss events between *SF3B1* WT and mutant samples. The column annotation at the top of the heatmap indicates different caner types. *z*-score represents normalized percent-spliced-in (PSI) values. (**D**) Volcano plot illustrating splicing changes of cryptic 3′ss events induced by mutant *SF3B1* between *SF3B1*^K700^ mutation and *SF3B1*^R625^ mutation in pan-cancer patient samples. Previously reported *SF3B1* mutation-induced 3′ss events are highlighted as yellow dots. (**E**) Schematic representation of the study design. (**F**) Hierarchical clustering and heatmap analysis of differentially spliced cryptic 3′ss events between *SF3B1* mutant and WT cell line samples. Rows and columns represent cryptic 3′ss events and cell line samples respectively. *z*-scores represents normalized PSI values. (**G**) Contingency table showing the number of cryptic 3′ss events preferentially associated with *SF3B1*^K700E^ mutation or *SF3B1*^R625H^ mutation in NHM and K562 cell lines. (**H** and **I**) Volcano plot depicting splicing changes of cryptic 3′ss events induced by mutant *SF3B1* between *SF3B1*^K700E^ mutation and *SF3B1*^R625H^ mutation in isogenic NHM (H) and K562 cells (I). (**J**) Integrative Genomics Viewer (IGV) plots showing representative cryptic 3′ss events preferentially induced by *SF3B1*^K700E^ or *SF3B1*^R625H^ mutations in isogenic NHM and K562 cells. (**K**) Reverse transcription polymerase chain reaction (RT-PCR) validation of representative cryptic 3′ss events preferentially associated with *SF3B1*^K700E^ or *SF3B1*^R625H^ mutations in isogenic NHM and K562 cells. Top colored dashes represent cryptic 3′ss usage, and bottom black dashes represent canonical 3′ss usage.

According to the COSMIC database, *SF3B1* hotspot mutations predominantly occur within HEAT repeat domains, with K700, K666, and R625 being the most frequently mutated residues (Fig. 1A). These mutations exhibited distinct prevalence on different cancer types. Among them, K700 mutations were primarily associated with hematologic malignancies. In contrast, R625 mutations are significantly enriched in melanoma, notably in UVM and SKCM (Fisher’s exact test, *P* < 0.0001) (Fig. 1B). This pattern aligns with observations reported in previous studies (*8*, *18*). This distinct enrichment of specific *SF3B1* mutational hotspots across different tumor types supports the hypothesis that mutation-specific splicing abnormalities may contribute to unique pathogenic mechanisms within each cancer lineage. To explore this possibility, we analyzed 111 RNA sequencing (RNA-seq) samples, including 37 hematologic tumors with K700 mutations, 18 melanomas with R625 mutations, and 58 *SF3B1* wild-type (WT) controls. This analysis was designed to uncover 3′ss defects in pre-mRNAs associated with different *SF3B1* mutation backgrounds across various cancer lineages (see Materials and Methods and table S1). Through unsupervised clustering analysis of the percent-spliced-in (PSI) values of cryptic 3′ss usage, we observed distinct splicing patterns between *SF3B1* R625 and K700 mutant (MUT) and matched WT samples (Fig. 1C). Specifically, the top 30 most significantly different splicing events (*P* < 0.05, MUT versus WT), ranked by the absolute ΔPSI (MUT versus WT), were summarized in table S2. Moreover, notable differences in aberrant 3′ss usage were detected between K700 and R625 mutations, independent of cancer type (Fig. 1C). We further compared the differences of aberrant 3′ss usage and found that many previously reported 3′ss events by *SF3B1* mutations, including *MAP3K7*, *PPP2R5A*, and *DLG1* (*8*, *18*, *26*), were differentially used between the two hotspots (Fig. 1D).

To investigate the underlying determinants of this preference, we engineered two cell models: NHMs to study *SF3B1* mutations in melanoma and K562 (a chronic myelogenous leukemia cell line) to model *SF3B1* mutations in hematologic malignancies (Fig. 1E). Each cell line was introduced with different *SF3B1* mutation types (WT/R625H/K700E) (Fig. 1E). Analysis of the locations of cryptic 3′splice sites in NHM and K562 cells revealed that they were located 10 to 24 nucleotides (nt) upstream of the respective canonical 3′ splice sites, consistent with previous studies in other cancer types (fig. S1A) (*6*, *7*, *18*). Principal components analysis (PCA) based on either the mRNA expression matrix or the PSI matrix of cryptic 3′ss events revealed that, compared to the mRNA expression matrix, the PSI matrix of cryptic 3′ss usage showed a clearer separation between *SF3B1*^K700E^, *SF3B1*^R625H^, and *SF3B1*^WT^ counterparts in both NHM and K562 lines (fig. S1B and tables S3 and S4). The result suggests that the influence of different *SF3B1* mutations on splicing status outweighs their impact on the overall gene expression level. Additionally, unsupervised clustering of PSI values for cryptic 3′ss events across all engineered NHM and K562 cells revealed clear separation in aberrant 3′ss usage between K700 and R625 mutations, regardless of cancer cell types (Fig. 1F). This pattern closely resembles the aberrant splicing observed in patient samples, as shown in Fig. 1C.

In addition to the differences in cryptic 3′ss between various *SF3B1* mutations, we also observed that, while the R625H and K700E mutations share some common aberrant splicing targets, each *SF3B1* mutation exhibits varying potency in missplicing the same cryptic 3′ss event. For most shared misspliced genes, the R625H mutation induced more cryptic 3′ss usage, as indicated by higher PSI values compared to K700E across different cell types Fig. 1F and table S3). Notably, within these shared misspliced events, a greater number of aberrant splicing events dominated by R625H were observed compared to K700E in both NHM and K562 cells (Fig. 1, G to I). Additionally, the splicing differences between these two mutations were significantly positively correlated across the two cell lines, suggesting that the observed differences are primarily caused by different *SF3B1* mutations rather than cell types (fig. S1C).

To experimentally validate the above findings, we performed reverse transcription polymerase chain reaction (RT-PCR) on the top differentially spliced genes between the R625H and K700E groups identified by mRNA data analysis. This included R625H-preferred transcripts (*CASD1*, *NF1*, *PLXNB1*, *SEPSECS*, and *KANSL3*) and K700E-preferred transcripts (*PPP2R3A*, *UBR4*, and *RNF2*), yielding consistent results (Fig. 1, J to K; and fig. S2, A to D). Together, above analysis identified specific preference of cryptic 3′ss changes between *SF3B1*^R625H^ and *SF3B1*^K700E^ mutations.

### Higher frequency of Poly-A in upstream of the cryptic AG site results in increased cryptic 3′ss usage by *SF3B1*^R625H^

The preceding results demonstrate that the *SF3B1*^R625H^ mutation exhibits a distinct preference for cryptic 3′ss events compared to the *SF3B1*^K700E^ mutation. To assess whether this pattern is influenced by gene expression levels across different cellular lineages, we categorized genes into three groups: those preferentially expressed in NHM cells, those preferentially expressed in K562 cells, and those equally expressed in both cell types (fig. S3A, outer circle). These gene groups were then separately analyzed for cryptic 3′ss events (fig. S3A, inner circle). We then checked the splicing preference on the genes in each of the groups (fig. S3A, inner circle). Notably, in all three gene groups, most of the cryptic 3′ss events exhibited higher PSI values on the *SF3B1*^R625H^ mutant than the *SF3B1*^K700E^. This observation indicates that the *SF3B1*^R625H^ mutation induces greater cryptic 3′ss splicing than *SF3B1*^K700E^, independent of cell lineage-specific gene expression.

**Fig. 2.**
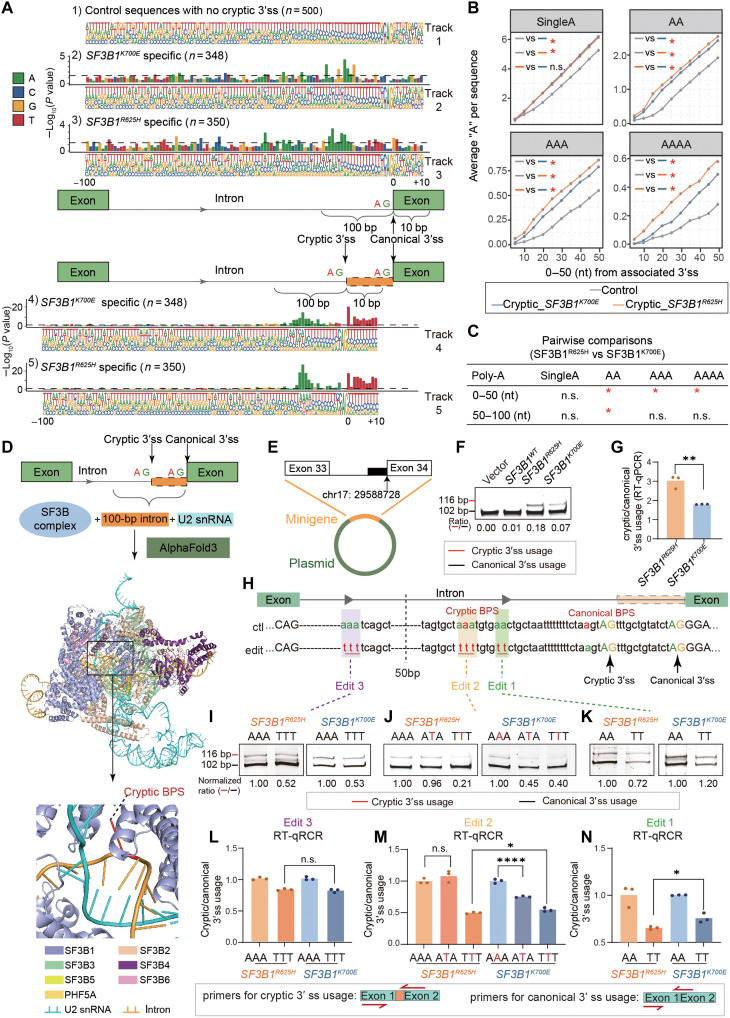
Poly-A enrichment upstream of cryptic AG sites enhances cryptic 3′ss usage by *SF3B1*^R625H^. (**A**) Consensus sequence motifs near canonical (tracks 2 and 3) and cryptic (tracks 4 and 5) AG splice sites. Track 1 represents control events without aberrant 3′ss usage. Sequence spans 100 nt upstream and 10 nt downstream of the AG site. Nucleotide size reflects frequency. Bar plots show –log_10_(*P* values) from Fisher’s exact tests versus controls (horizontal dashed line, *P* = 0.05). (**B**) Average frequencies of motif features (“A,” “AA,” “AAA,” and “AAAA”) within 50 bp upstream of cryptic AG site preferentially used by *SF3B1*^K700E^ or *SF3B1*^R625H^ mutations, as well as control AG sites without upstream aberrant 3′ss usage. Statistical significance is indicated. (**C**) Pairwise comparisons of motif feature frequencies between *SF3B1*^K700E^ and *SF3B1*^R625H^ mutations using the Wilcoxon signed-rank test with continuity correction. (**D**) Alphafold3 predicted cryptic branch point for *SF3B1*^R625H^-mediated *NF1* missplicing. The top section presents the schematic diagram of predicted protein-RNA interactions, and the bottom section displays the prediction results, with the predicted branch point adenine shown as red stick. (**E**) Schematic diagram of the *NF1* minigene plasmid. (**F** and **G**) RT-PCR (F) and RT–quantitative polymerase chain reaction (qPCR) (G) validation of differential cryptic 3′ss usage between *SF3B1*^K700E^ and *SF3B1*^R625H^ in isogenic 293 T cells. (**H**) Sequences flanking the canonical and cryptic AG splice sites in the unedited *NF1* minigene (top) and edited *NF1* minigenes (bottom). Edited positions are labeled as edits 1, 2, and 3. (**I** to **K**) RT-PCR results showing cryptic and canonical 3′ss usage in *NF1* minigenes with specific edits (edit 1, 2, or 3). (**L** to **N**) RT-qPCR analysis of cryptic and canonical 3′ss usage in 293 T cells with different edited *NF1* minigenes. The *y* axis represents cryptic/canonical 3′ss ratio. Statistical significance by *t* test (n.s., not significant; *P < 0.05; **P < 0.01; ****P < 0.0001).

*SF3B1* is a core component of the U2 small nuclear ribonucleoprotein (snRNP) complex, which plays a crucial role in BPS recognition and spliceosome assembly at upstream 3′sss during the early stages of splicing. As such, conserved sequences upstream of the 3′ss, including the AG site, polypyrimidine (poly-Py) tract (comprising T and C), and BPS, are essential for correct splicing. To investigate any specific change on these key intronic sequence features, we next analyzed sequence motifs associated with misspliced events of different *SF3B1* hotspot mutations. For this comparison analysis, we selected the top 350 events favoring *SF3B1*^R625H^, 348 cryptic 3′ss events favoring *SF3B1*^K700E^, and 500 canonical 3′sss without cryptic 3′ss switch as controls (Fig. 2A; see Materials and Methods). Motif analysis was performed on sequences spanning 100 nt upstream and 10 nt downstream the cryptic and associated canonical 3′ss. The upstream of the canonical 3′ss exhibit a typical poly-Py tract, characterized by more than 20 base pairs (bp) of T- and C-enriched sequences (Fig. 2A, track 1), which is consistent with previous reports (*27*). In contrast, we observed a shortened and weaker poly-Py tract, along with an enrichment of poly-A upstream of the cryptic 3′ splice sites induced by *SF3B1* mutations (Fig. 2A, tracks 4 and 5), which is clearer and more distinct than those of the canonical 3′ site (Fig. 2A, tracks 2 and 3). This pattern has been previously reported for *SF3B1*^K700E^ mutation-associated misspliced sequences (*7*, *16*). Furthermore, unexpectedly, we observed a significantly higher enrichment of poly-A sequences (AAAA) upstream of the R625H-specific cryptic 3′sss compared to the K700E group (Fig. 2A, tracks 4 and 5). We also enriched six-mer motifs in 30 bp upstream of the cryptic/canonical AG site (fig. S3B), revealing that T and C were the most frequent nucleotides in the control. In the R625H group, the second most frequent sequence was a continuous stretch of A’s, while, in the K700E group, an additional C followed five consecutive A’s. This finding suggests that the higher enrichment of poly-A sequences may correlate with an increased preference for R625H-specific cryptic 3′ss usage. To further quantify this poly-A differences, we calculated the averaged occurrence of consecutive A’s (single A, AA, AAA, and AAAA) in the 100 bp upstream of the cryptic and canonical AG sites (Fig. 2B and fig. S3C). Consistent with the motif analysis, we found that *SF3B1* mutant sequences exhibited a higher enrichment of poly-A compared to the control sequences. In addition, a significantly higher occurrence of consecutive A’s (AA, AAA, and AAAA) exists upstream the R625H-specific cryptic 3′ss compared to K700E, particularly within the first 50 bp (Fig. 2C).

Given these results, we hypothesized that poly-A sequences located within the first 50 bp upstream of the cryptic AG site influence the preference of cryptic 3′ss activation in *SF3B1*^R625H^ compared to *SF3B1*^K700E^ mutant cells. Because BPS is commonly located within 50 bp upstream of the splice site (*28*), we then hypothesized that the observed features might be associated with the sequence characteristics of the BPS. To determine whether these poly-A elements are positioned near the cryptic BPS, we used AlphaFold3 to predict cryptic branch point adenines within the complete SF3B complex and its associated intronic sequences. Our analysis identified a cryptic branch point adenine located 31 nt upstream of the cryptic 3′ss (Fig. 2D and fig. S3, D and E). Notably, this cryptic branch point adenine was embedded within an AAA sequence, with the middle “A” serving as the cryptic branch point adenine, further confirming that the enriched poly-A is within and surrounding the cryptic BPS.

To experimentally validate whether poly-A sequences near the cryptic BPS determine the missplicing preference of *SF3B1*^R625H^ versus *SF3B1*^K700E^, we engineered a minigene spanning exon 33 and exon 34 of *NF1* pre-mRNA (Fig. 2E). This splice site is aberrantly activated in all *SF3B1* hotspot mutants, with significantly higher PSI values in *SF3B1*^R625H^ compared to *SF3B1*^K700E^ (Fig. 2, F and G). Using site-directed mutagenesis, we modified poly-A sequences within or surrounding the cryptic BPS within the minigene to evaluate their impact on splicing preference, using canonical BPS mutagenesis as reference. As expected, A to T for the canonical branching adenine completely blocked canonical splicing (fig. S3, F to H). Mutation of the cryptic branch point adenine alone (edit 2) did not significantly affect cryptic splicing in *SF3B1*^R625H^ cells (Fig. 2, J and M; and fig. S3, J and M). However, in *SF3B1*^K700E^ cells, the same mutation led to a pronounced reduction in cryptic splicing (Fig. 2, J and M; and fig. S3, J and M). This difference suggests that *SF3B1*^R625H^ can use surrounding adenines as alternative branch point adenine, thereby enhancing its ability to engage in cryptic splicing. When the “AAA” sequences at the branch point adenine are all mutated to “TTT” (edit 2), *SF3B1*^R625H^ exhibited complete loss of cryptic splicing, likely due to the absence of functional cryptic branch point adenine or alternative branch point adenine in close proximity. These findings suggest that poly-A enrichment at the cryptic branch point adenine enhances *SF3B1*^R625H^-mediated cryptic splicing by providing alternative branch points, thereby increasing the likelihood of cryptic splice site activation compared to *SF3B1*^K700E^.

To further assess whether poly-A sequences surrounding the cryptic branch point adenine contribute to *SF3B1*^R625H^-driven missplicing, we mutated an upstream “AA” dinucleotide located 5 bp from the cryptic branch point adenine to “TT” (edit 1). This modification resulted in a more substantial reduction in cryptic splicing in *SF3B1*^R625H^ than in *SF3B1*^K700E^, as confirmed by DNA gel electrophoresis and RT–quantitative polymerase chain reaction (qPCR) (Fig. 2, K and N; and fig. S3, K and N). In contrast, mutating poly-A sequences 50 to 100 bp upstream of the cryptic AG site, which located more than 50 bp from the cryptic branch point adenine (edit 3), had no notable effect on cryptic splicing in either *SF3B1*^K700E^ or *SF3B1*^R625H^ cells (Fig. 2, I and L; and fig. S3, I and L). These findings confirm that poly-A sequences at and immediately surrounding the cryptic branch point adenine serve as key determinants of *SF3B1*^R625H^*-*driven missplicing, providing an intrinsic advantage over *SF3B1*^K700E^ in the selection of cryptic 3′ss.

### *SF3B1*^R625H^ mediates stronger RAS activation in melanoma

**Fig. 3.**
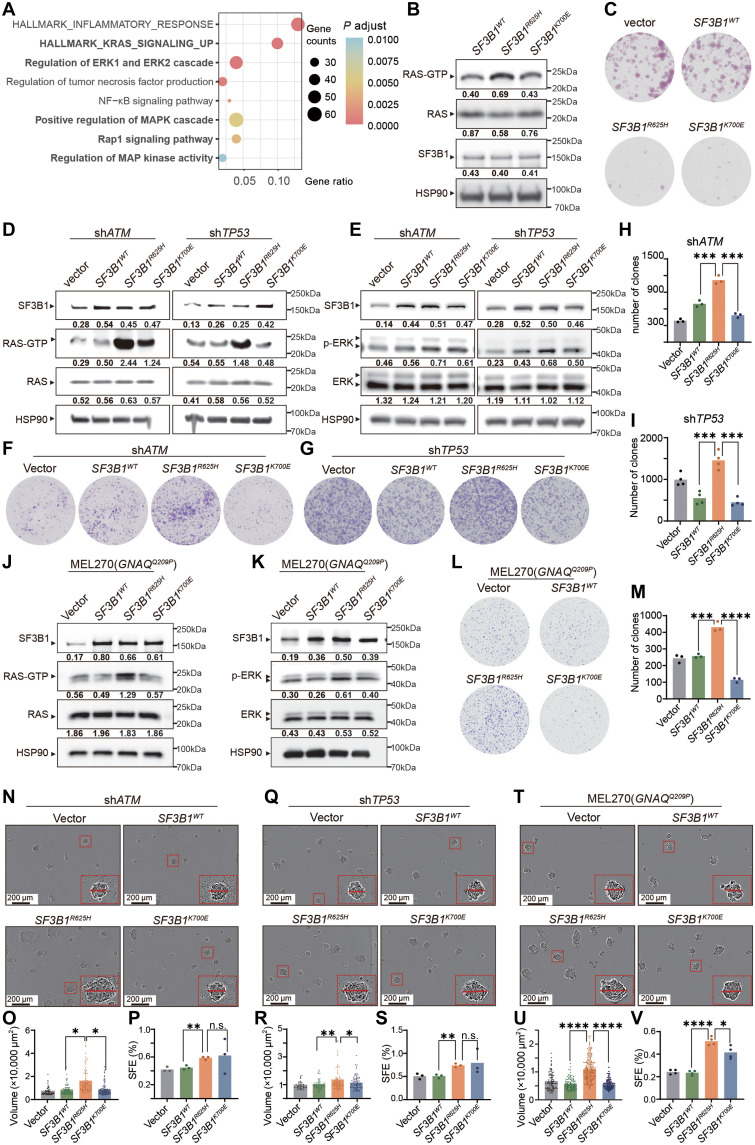
*SF3B1*^R625H^ mutation mediates stronger RAS activation than *SF3B1*^K700E^ mutation. (**A**) Bubble plot depicting pathway enrichment analysis of differentially expressed genes between *SF3B1*-WT and *SF3B1*-mutant melanoma tumors from TCGA (The Cancer Genome Atlas) data. The *x* axis indicates gene ratio, and the *y* axis shows enriched pathways. Bubble size reflects gene count, and the color gradient represents adjusted *P* value. NF-κB, nuclear factor κB. (**B**) Western blot analysis of RAS activity [RAS–guanosine 5-triphosphate (GTP) level] in MUM2B melanoma cells overexpressing indicated *SF3B1* mutations. Quantitative analyses normalized to HSP90 are labeled below related band. (**C**) Colony formation assay comparing MUM2B cells expressing empty vector or *SF3B1* variants. (**D**) RAS activation in MUM2B cells with *SF3B1* mutations under *ATM* or *TP53* knockdown. (**E**) Western blot detection of MAPK pathway activation in MUM 2B cells with *SF3B1* mutations in the context of *ATM* or *TP53* knockdown. (**F** and **G**) Colony formation assays of MUM2B cells expressing *SF3B1* variants with *ATM* (F) or *TP53* (G) knockdown. (**H** and **I**) Quantification of colony formation in (F) and (G). (**J**) RAS activation assay in MEL270 cells (with *GNAQ*^Q209P^ mutation) expressing *SF3B1* variants. (**K**) Western blot detection of MAPK pathway activation in MEL270 cells expressing *SF3B1* variants. (**L** and **M**) Colony formation (L) and quantification (M) of MEL270 cells expressing *SF3B1* variants. (**N** to **P**) Sphere formation of MUM2B cells expressing *SF3B1* variants with ATM knockdown. *n* = 3; scale bars, 200 μm. Quantifications of sphere volume (O) and sphere formation efficiency (SFE) (P). SFE reflects the percentage of spheres formed per seeded cells. (**Q** to **S**) Sphere formation of MUM2B cells expressing *SF3B1* variants with *TP53* knockdown. *n* = 3; scale bars, 200 μm. Quantifications of sphere volume (R) and sphere formation efficiency (S). (**T** to **V**) Sphere formation of MEL270 cells expressing *SF3B1* variants. *n* = 3; scale bars, 200 μm. Quantifications of sphere volume (U) and sphere formation efficiency (V). Statistical significance by *t* test (n.s., not significant; *P < 0.05; **P < 0.01; ***P < 0.001; ****P < 0.0001).

As shown in Fig. 1B, mutations at position R625 residue of *SF3B1* occur predominantly in melanoma (*13*–*15*). To understand the role the *SF3B1* mutation in promoting melanoma tumorigenesis, we performed functional enrichment analysis based on the differentially expressed genes between *SF3B1* mutant and WT samples of The Cancer Genome Atlas (TCGA) melanoma cohort (table S4). This analysis revealed that inflammatory response pathways and RAS associated pathways [RAF–mitogen-activated protein kinase (MAPK) kinase (MEK)–extracellular signal–regulated kinase (ERK)] were top enriched (Fig. 3A). Given the established role of MAPK pathway activation in melanoma progression, commonly driven by activating mutations in *NRAS* and *BRAF* (*29*), this finding suggested a potential link between *SF3B1*^R625^ mutation and RAS-MAPK pathway activation. To check whether *SF3B1*^R625H^ mediates MAPK activating by direct activating RAS, Ras activity was evaluated in melanoma cells overexpressing different *SF3B1* variants (*SF3B1*^WT^, *SF3B1*^R625H^, or *SF3B1*^K700E^). Notably, cells overexpressing *SF3B1^R625H^* exhibited stronger RAS activation compared to *SF3B1*^K700E^ cells (Fig. 3B). However, melanoma cell growth was suppressed when mutant *SF3B1* variants were expressed isogenically (Fig. 3C and fig. S4A), likely because mutant *SF3B1* exacerbates aberrant splicing events, leading to widespread misprocessing of essential transcripts and cellular stress. These growth-inhibitory effects indicate that additional genetic alterations are required to tolerate mutant *SF3B1*–induced splicing stress and unmask its tumor-promoting potential. *ATM* loss and *TP53* mutations frequently co-occur with *SF3B1* mutations in CLL (fig. S4B), while *ATM* loss is also observed in *SF3B1*-mutant UVM (fig. S4C). These contexts were used to model genetic environments that permit tolerance of mutant *SF3B1*-induced splicing stress (*30*). Meanwhile, *ATM* loss has been shown to synergize with *SF3B1* mutations in promoting hematologic malignancies (*31*), suggesting a potential cooperative role in melanoma as well. Therefore, we cotransfected cells with short hairpin RNAs (shRNAs) targeting *ATM* and *TP53* (fig. S5, A and B) to enhance cellular tolerance to the isogenic expression of mutant *SF3B1*. Consistently, in the context of *ATM* or *TP53* knockdown, *SF3B1*^R625H^ cells demonstrated robust RAS activation compared to *SF3B1*^WT^ cells, whereas *SF3B1*^K700E^ cells showed less pronounced activation (Fig. 3D). In addition, *SF3B1*^R625H^ showed stronger activation the RAS downstream MAPK signaling pathway than *SF3B1*^K700E^ cells as indicated by increased ERK phosphorylation (Fig. 3E).

To explore whether melanoma cells with the *SF3B1*^R625H^ gain growth advantage over those with the *SF3B1*^K700E^ cells, we overexpressed either the *SF3B1*^R625H^ or *SF3B1*^K700E^ in the triple-WT (*BRAF*/*NRAS*/*NF1* WT) MUM2B, a commonly used UVM cell line (*32*). *SF3B1*^R625H^ cells displayed a significant increase in colony formation compared to *SF3B1*^K700E^ cells (Fig. 3, F to I; and fig. S5, C and D). As *SF3B1* and *GNAQ* mutations frequently co-occur in UVM (fig. S6A), we further expressed different *SF3B1* variants in MEL270 cells, a UVM line harboring the *GNAQ*^Q209P^ mutation (Fig. 3, J to M, and fig. S6B). Consistent with the *ATM* or *TP53* knockdown in MUM2B cells, *SF3B1*^R625H^ mutated MEL270 cells showed stronger activation of RAS/MAPK pathway (Fig. 3, J and K) and promoted greater colony formation and larger tumor spheroid growth than their *SF3B1*^K700E^ cells (Fig. 3, L to V). Collectively, these findings demonstrate that the *SF3B1*^R625H^ mutation preferentially activates the RAS/MAPK pathways and promotes melanoma cell growth, highlighting its role in driving melanoma progression across diverse cooperating genetic contexts.

### *SF3B1*^R625H^ mutation promotes RAS activation through aberrant splicing of *NF1*

**Fig. 4.**
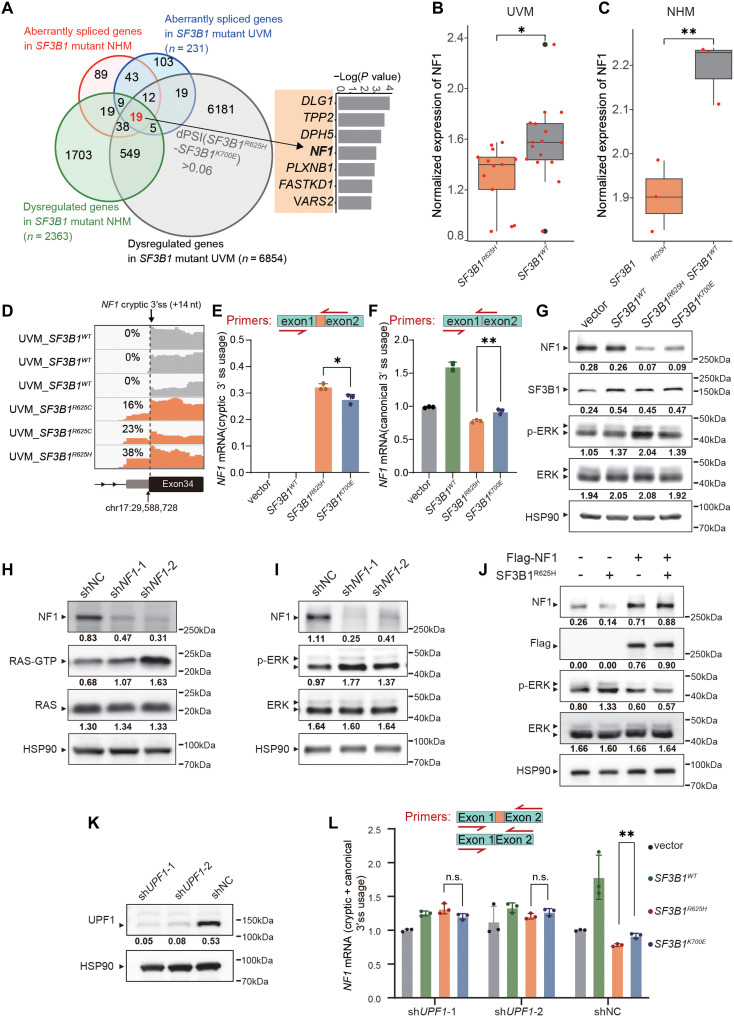
*SF3B1*^R625H^ mutation promotes RAS activation through aberrant splicing of *NF1*. (**A**) Venn diagrams showing the significantly differentially expressed and spliced genes between *SF3B1* mutant versus WT samples across UVM tumors from TCGA and NHM cell line (left). The overlapping genes are listed with corresponding *P* values for differential splicing (right). (**B** and **C**) *NF1* mRNA expression levels (log_2_ RPKM) in *SF3B1* mutant versus WT samples from patients with UVM in the TCGA cohort (B) and NHM cells (C). Thick bars represent median values (*P < 0.05; **P < 0.01). (**D**) IGV plots of *NF1* cryptic 3′ss events in patients with UVM (TCGA cohort) with or without *SF3B1* mutations. (**E**) RT-qPCR quantification of aberrantly spliced *NF1* mRNA in isogenic MUM2B melanoma cells with the indicated *SF3B1* mutations (statistical significance by *t* test, *P < 0.05). (**F**) RT-qPCR quantification of canonically spliced *NF1* mRNA in isogenic MUM2B melanoma cells with the indicated *SF3B1* mutations (statistical significance by *t* test, **P < 0.01). (**G**) Western blot analysis of *NF1* protein levels and ERK phosphorylation status in isogenic MUM2B cells expressing WT or mutant *SF3B1*. Quantitative analyses normalized to HSP90 are labeled below related band. (**H**) RAS activation assay in MUM2B cells following *NF1* knockdown. (**I**) Western blot detection of MAPK signaling pathway activation, indicated by phosphorylated ERK (p-ERK), in MUM2B cells with *NF1* knockdown. (**J**) Western blot detection of MAPK signaling pathway activation in MUM2B cells with *NF1* overexpression. (**K**) Western blot validation of *UPF1* knockdown efficiency using shRNA. (**L**) RT-qPCR quantification of total *NF1* mRNA levels with or without *UPF1* knockdown in isogenic cells expressing WT or mutant *SF3B1*. *P* values are calculated by *t* test (*n* = 3; n.s., not significant; **P < 0.01).

To elucidate how the *SF3B1*^R625H^ mutation preferentially drives RAS activation, we screened candidate targets based on differentially spliced and expressed genes in *SF3B1* mutant UVM and NHM samples (tables S5 and S6). This analysis identified a set of misspliced RAS regulatory genes associated with the *SF3B1* mutation, with *NF1* standing out as a key target (Fig. 4A). *NF1* encodes neurofibromin, a well-established negative regulator of RAS, which modulates RAS activity by stimulating its guanosine triphosphatase function, converting RAS from its active guanosine 5-triphosphate–bound form to its inactive GDP-bound form.

While *NF1* missplicing has been reported in *SF3B1*^K700E^ tumors and isogenic 293 T cells, whether the *SF3B1*^R625H^ mutation induces similar aberrant splicing and consequent effect on RAS activity remained unclear (*33*). Bulk mRNA sequencing data analysis showed that, in addition to aberrant *NF1* splicing, *NF1* mRNA levels was markedly reduced in *SF3B1^R625H^* mutant UVM tumor samples and NHM cells (Fig. 4, B to D). Based on this, we hypothesized that the *SF3B1*^R625H^ mutation activates RAS by aberrant splicing of *NF1* pre-mRNA and mRNA degradation, leading to the dysregulation of its negative regulatory function.

RT-PCR analysis revealed increased cryptic 3′ss usage in *NF1* pre-mRNA in *SF3B1*^R625H^ melanocytes compared to *SF3B1*^K700E^ cells. Additionally, RT-qPCR confirmed more extensive aberrant splicing in the *SF3B1*^R625H^ mutation (Fig. 4E), accompanied by a significant reduction in normal *NF1* mRNA levels compared to *SF3B1*^K700E^ (Fig. 4F). Consistent with these findings, Western blotting showed a marked reduction in NF1 protein, with corresponding MAPK pathway activation evidenced by elevated ERK phosphorylation (Fig. 4G). Additionally, *NF1* knockdown via shRNA mimicked the RAS and MAPK pathway activation seen in *SF3B1^R625H^* cells, underscoring *NF1* as the primary mediator of *SF3B1*^R625H^-driven RAS activation (Fig. 4, H and I), whereas restoration of NF1 expression effectively suppressed *SF3B1*^R625H^-induced MAPK activation (Fig. 4J). Collectively, these results identify *NF1* as the key mediator of *SF3B1*^R625H^-driven RAS activation.

In addition to *NF1*, we identified another RAS negative regulator, *RASA1*, as a missplicing target of *SF3B1* mutations in both NHM and K562 cells. RT-PCR revealed enhanced cryptic 3′ss usage in *RASA1* pre-mRNA, particularly in *SF3B1*^R625H^ compared to *SF3B1*^K700E^ cells (fig. S7, A and B). Consistently, Western blot analysis showed a greater reduction in RASA1 protein level in *SF3B1*^R625H^-overexpressed melanoma cells (fig. S7C). Functionally, *RASA1* knockdown elevated RAS activity and ERK phosphorylation, although less strongly than *NF1* knockdown (fig. S7D). Codepletion of *NF1* and *RASA1* produced additive activation of RAS and MAPK signaling (fig. S7D). These findings indicate that *SF3B1*^R625H^-driven RAS activation arises from combined missplicing of multiple RAS inhibitors, with *NF1* serving as the dominant contributor.

To further understand how *NF1* missplicing leads to reduced *NF1* expression, we analyzed the sequence of misspliced *NF1* transcript. *SF3B1*^R625H^-driven cryptic 3′ss introduced a 14-nt intronic insertion, resulting in a premature termination codon that likely triggers NMD. To test this, we knocked down *UPF1*, a key regulator of NMD (Fig. 4K), in *SF3B1*^R625H^ cells. This intervention restored *NF1* mRNA levels that were, otherwise, reduced by the *SF3B1*^R625H^ mutation, confirming that *UPF1*-driven NMD is the primary mechanism underlying the *SF3B1*^R625H^-mediated decrease in *NF1* expression (Fig. 4L).

### *SF3B1^R625H^* mutation drives melanoma progression via RAS activation

**Fig. 5.**
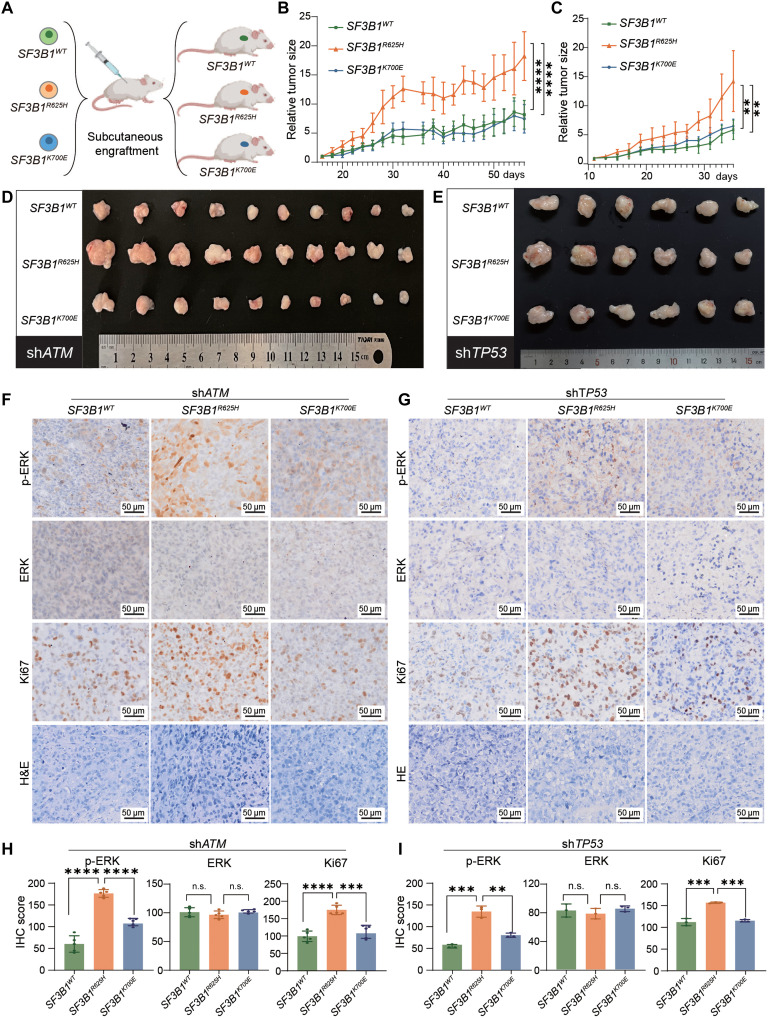
*SF3B1*^R625H^ mutation promotes melanoma development via RAS activation. (**A**) Schematic representation of the generation of MUM2B cell-derived xenograft (CDX) models with different *SF3B1* mutation status. (**B**) Evaluation of tumor volume changes in the MUM2B CDX models with different *SF3B1* mutation status in the context of *ATM* knockdown. *n* = 10; ****P < 0.0001. (**C**) Evaluation of tumor volume changes in the MUM2B CDX models with different *SF3B1* mutation status in the context of *TP53* knockdown. *n* = 6; **P < 0.01. (**D** and **E**) Images of the tumor with different *SF3B1* mutations in the context of *ATM* (D) or *TP53* (E) knockdown. (**F** and **G**) Immunohistochemical staining of tumors in (D) and (E) to evaluate protein status of phosphorylated ERK, total ERK, and Ki67. (**H** and **I**) Immunohistochemical score from (F) and (G). *P* values are calculated by *t* test (n.s., no significance; **P < 0.01; ***P < 0.001; ****P < 0.0001).

To assess whether the *SF3B1^R625H^* mutation promotes melanoma progression, we evaluated tumor growth in MUM2B melanoma cells harboring *SF3B1*^WT^, *SF3B1*^R625H^, or *SF3B1*^K700E^, in the context of *ATM* or *TP53* knockdown to facilitate tumor growth (Fig. 5A). As anticipated, melanoma cells harboring the *SF3B1*^R625H^ mutation exhibited significantly enhanced tumor growth compared to those with *SF3B1*^K700E^ or *SF3B1*^WT^, as demonstrated by increased tumor volume and weight (Fig. 5, B to E; and fig. S8, A and B). Immunohistochemical staining further revealed a higher Ki67 proliferation index in *SF3B1*^R625H^ tumors, indicating a proliferation advantage conferred by this mutation (Fig. 5, F to I). Additionally, the *SF3B1*^R625H^ mutation induced robust activation of the MAPK pathway, as evidenced by markedly elevated ERK phosphorylation in immunohistochemical analyses (Fig. 5, F to I). These findings reinforce the role of the *SF3B1*^R625H^ mutation in driving melanoma growth through RAS-MAPK pathway activation.

**Fig. 6.**
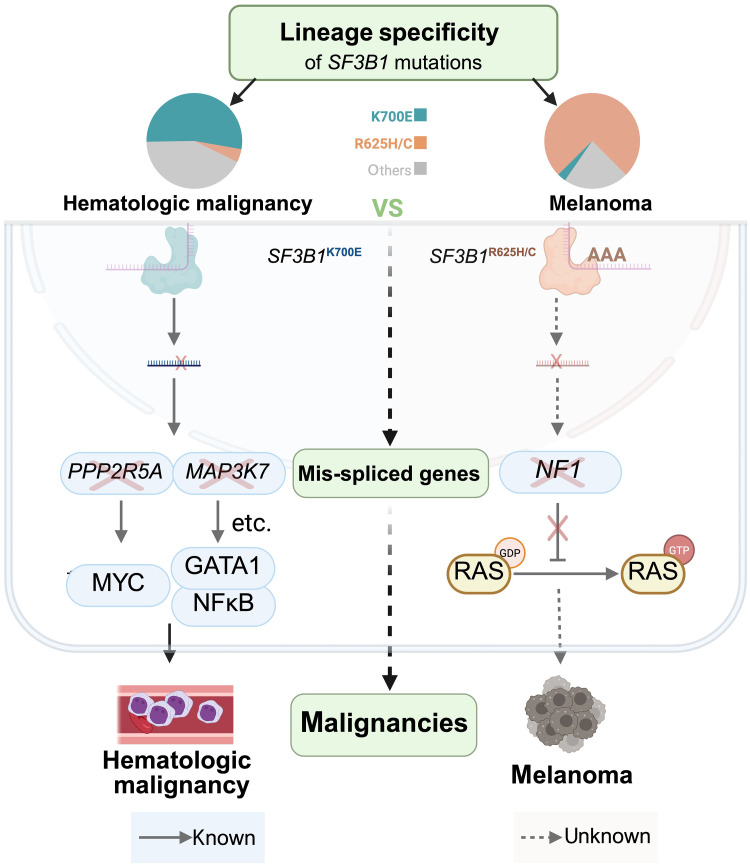
Mechanistic model of *SF3B1*^R625^ mutation–driven melanoma tumorigenesis. The *SF3B1^R625^* mutation promotes RAS activation through aberrant splicing of *NF1*, thereby driving melanoma tumorigenesis.

In summary, our in vitro analyses demonstrate that, compared to *SF3B1*^K700E^, the *SF3B1*^R625H^ mutation induces a greater degree of missplicing in RAS negative regulators, primarily *NF1*, thereby enhancing RAS pathway activation. Consistently, *SF3B1*^R625H^ expression significantly accelerated melanoma growth in xenograft models, aligning with its stronger impact on RAS-MAPK signaling (Fig. 6).

## DISCUSSION

The melanocyte-specific occurrence of the *SF3B1*^R625^ mutation, and its absence in other cancers, has long posed an intriguing question about lineage-restricted oncogenic mechanisms. Melanoma initiation is frequently driven by MAPK pathway–activating mutations, such as *NRAS*^Q61R^, *BRAF*^V600E^, or *GNAQ* mutations, yet whether *SF3B1*^R625^ mutation plays a role in MAPK pathway activation has remained unclear. In this study, we reveal that *SF3B1*^R625^ mutation directly activates the RAS-MAPK pathway by selectively inducing *NF1* pre-mRNA missplicing, leading to *NF1* mRNA degradation via NMD. This splicing-driven mechanism not only activates the MAPK pathway but also establishes a clear role for *SF3B1*^R625^ mutation in melanocyte-specific tumorigenesis, offering previously unaddressed insight into its lineage-restricted oncogenic potential.

We and others have shown the distinct aberrant splicing patterns associated with different *SF3B1* hotspot mutations (*8*, *34*), but the driving mechanism remains unclear. In our study, we discovered that *SF3B1*^R625H^ can more efficiently use the poly-A’s surrounding the branch point as alternative branch point adenine, thereby facilitating cryptic splicing. We also discovered that *SF3B1*^R625^ mutation gains a missplicing advantage over *SF3B1*^K700E^ specifically in genes with poly-A enrichment 0 to 50 bp upstream of the cryptic AG site. This sequence-specific preference allows *SF3B1*^R625^ mutation to strongly and selectively missplice genes with this unique feature, providing a molecular basis for its role in lineage-specific tumorigenesis when the targeted genes are essential in a particular cancer type, such as melanoma. This linear progression, from sequence preference to gene selection to cancer specificity, offers a compelling mechanistic explanation for the selectivity of *SF3B1* hotspot mutations across different cancers.

The molecular mechanisms underlying the greater enrichment of poly-A sequences near cryptic BPSs compared to canonical BPS remain unresolved. In splicing, WT SF3B1 ensures high-fidelity recognition of canonical BPS by interacting with U2 snRNP, which binds tightly to the BPS to facilitate accurate splice site selection. However, *SF3B1*^R625^ mutation induces a shift in branch point adenines recognition, leading to the preferential activation of cryptic BPS. A key molecular factor driving this shift is the role of pseudouridine (Ψ) within U2 snRNA (*35*). Ψ enhances base-pairing stability, particularly with adenosine (A), forming a more stable Watson-Crick Ψ-A pair compared to the standard U-A pair (*36*, *37*). This increased stability alters the interaction landscape of U2 snRNA, making poly-A–enriched sequences more favorable for U2 snRNA binding. Because Ψ-A pairings provide greater stability, cryptic branch point adenine surrounded by poly-A tracts creates a more stable platform for U2 snRNA binding, thereby increasing the likelihood of cryptic splicing. In contrast, canonical BPs, which are recognized with higher fidelity by WT SF3B1, do not require such stabilizing interactions. While this provides a mechanistic rationale for the enrichment of poly-A sequences near cryptic BPs, a more detailed structural explanation is required to fully elucidate how *SF3B1*^R625^ mutations alter U2 snRNA–BP interactions at the atomic level. Future studies incorporating structural modeling and cryo–electron microscopy analysis may help resolve these mechanistic details.

Our findings suggest that the preferential occurrence of the *SF3B1*^R625^ mutation in melanoma may be attributed to its more potent induction of RAS activation. However, cryptic 3′ss analysis of bulk mRNA sequencing data from *SF3B1*^R625^-mutated cells and samples from patients with melanoma revealed a wide array of aberrantly spliced genes, indicating the involvement of additional factors beyond *NF1* in melanoma tumorigenesis. Mechanisms previously associated with *SF3B1*^K700E^ mutations, such as *BRD9* missplicing (*17*) and MYC protein stabilization (*8*), may similarly contribute to melanoma progression driven by *SF3B1*^R625^ mutations. We further propose that other genes or co-occurring mechanisms likely play essential roles in the oncogenic effects of the *SF3B1*^R625^ mutation. Elucidating how this extensive list of aberrantly spliced genes cooperates to drive melanoma tumorigenesis represents a critical avenue for future investigation.

Because *SF3B1* mutations are typically heterozygous in cancer (*38*, *39*), reflecting the necessity to retain one functional allele for constitutive pre-mRNA splicing, CRISPR-based generation of cells harboring a heterozygous *SF3B1* mutation would be the ideal approach to preserve the physiological balance between mutant and WT SF3B1 expression. However, establishing such precise knock-in models is technically challenging, as primary human melanocytes and MUM2B melanoma cells in our system are refractory to efficient transfection and genome editing. Moreover, given that *SF3B1* is an essential splicing factor, its complete loss or homozygous mutation severely compromises cell viability (*40*). Nevertheless, overexpression and isogenic systems remain informative because both WT and mutant SF3B1 proteins coexist and are functionally expressed. Isotopic expression of *SF3B1* hotspot mutations consistently alter branch-point selection and induce cryptic 3′ss usage observed in patient tumors (*8*, *24*). Prior studies confirm that such models faithfully reproduce mutant-associated splicing defects (*34*). Thus, while CRISPR knock-in would best capture allele-specific physiology, our overexpression approach, supported by tumor RNA-seq and functional rescue data, demonstrates that *SF3B1*^R625H^, more than *SF3B1*^K700E^, drives cryptic splicing of RAS regulators and promotes melanoma growth.

Translating our findings into clinical management for *SF3B1*-mutated melanoma presents an important direction for future study. Our findings provide mechanistic insight into why melanoma preferentially harbors the *SF3B1*^R625^ mutation and may help guide the development of new therapeutic strategies tailored to *SF3B1*^R625^-driven melanoma growth. Targeting the RAS-MEK pathway may represent a potential strategy for treating *SF3B1*^R625^-mutant melanoma as this mutation activates RAS/MEK pathway. However, direct evidence demonstrating enhanced sensitivity of these *SF3B1*^R625^-mutant cells to MEK inhibitors is now lacking. Moreover, *NF1* loss, a downstream consequence of *SF3B1* mutation, is a well-recognized mechanism of resistance to combined BRAF and MEK inhibition in *BRAF*^V600E^-mutated melanoma (*41*, *42*). Because NF1 normally suppresses RAS activity, its loss leads to sustained RAS signaling and activation of alternative survival pathways, such as the phosphatidylinositol 3-kinase (PI3K)–Akt pathway (*43*), thereby contributing to therapeutic resistance. Therefore, MEK inhibition alone may be insufficient to effectively target *SF3B1*-mutated melanoma. A more effective approach may involve combining MEK inhibition with PI3K pathway inhibition or developing strategies such as pan-RAS inhibitors or drugs and PROTACs specifically targeting the SF3B1^R625^ mutant protein or its associated complexes. These approaches may provide a more comprehensive strategy for eradicating *SF3B1*^R625^ mutant melanomas.

## MATERIALS AND METHODS

### Cell lines

NHMs (ScienCell, no. 2200), as well as K562, MUM2B, and human embryonic kidney (HEK) 293T cell lines (obtained from the China Center for Type Culture Collection, Wuhan, China), were used in this study. NHM cells were cultured in Melanocyte Medium (ScienCell, no. 2201), supplemented with 1% melanocyte growth supplement (ScienCell, no. 2252), 0.5% fetal bovine serum (FBS; ScienCell, no. 0002), and 1% penicillin/streptomycin (ScienCell, no. 0503). K562 cells were maintained in RPMI 1640 medium (Gibco, no. 11875500BT) with 10% FBS (Gibco, no. 10270-106). MUM2B and HEK293T cells were cultured in Dulbecco’s modified Eagle’s medium (DMEM, Gibco, no. 11885500BT) with 10% FBS (Gibco, no. 10270-106). All cell cultures were incubated at 37°C with 5% CO_2_.

### Plasmids

To overexpress various *SF3B1* variants in human cells, the *SF3B1* cDNA sequence was optimized with synonymous mutations to minimize toxicity during bacterial expansion and to differentiate exogenous from endogenous *SF3B1* sequences. The modified *SF3B1* cDNA was then cloned into the pLVX lentiviral vector tagged with green fluorescent protein (GFP) for visualization. Point mutations R625H and K700E were introduced into the *SF3B1* plasmid using the QuickMutation Site-Directed Mutagenesis Kit (Beyotime, no. D0206).

shRNAs targeting *NF1*, *ATM*, *TP53*, and *UPF1* were cloned into the pSLenti-U6-shRNA vector. The specific shRNA sequences used are listed. shNF1: TGCGCAGTTAGCAGTTATAAA (#1); shNF1: TAAGCGGCCTCACTACTATTT (#2). shATM: TGGTCAAATACTTCATCAAAT. shTP53: CACCATCCACTACAACTACAT. shUPF1: AGATATGCCTGCGGTACAAAG (#1); shUPF1: TTACCTTGGTGACGAGTTTAA (#2). shRASA1: CAGCTCCCATATACCATTAAA.

### Bulk RNA-seq

Lentiviruses were prepared in HEK293T cells and used to infect NHM and K562 cells. Cells stably expressing *GFP* and different *SF3B1* variants were selected 72 hours after infection. Cells (1 × 10^5^) were collected for each sample. Cells were lysed with TRIzol reagent (Thermo Fisher Scientific, no. 15596026) immediately and store at −80°C for total RNA isolation.

The RNA samples for bulk mRNA-seq were placed in RNAlater (Thermo Fisher Scientific, AM7020) and stored in liquid nitrogen before sending to Novogene Bioinformatics Technology Co. Ltd. (Beijing, China) to conduct mRNA sequencing. Total RNA was used as input material for the RNA sample preparations. Sequencing libraries were generated using the NEBNext Ultra RNA Library Prep Kit for Illumina [New England Biolabs (NEB), USA, catalog no. E7530L] following the manufacturer’s recommendations, and index codes were added to attribute sequences to each sample. Briefly, mRNA was purified from total RNA using poly-T oligo-attached magnetic beads. Fragmentation was carried out using divalent cations under elevated temperature in NEBNext First Strand Synthesis Reaction Buffer (5×). First-strand cDNA was synthesized using random hexamer primer and M-MuLV Reverse Transcriptase [ribonuclease H (RNase H)]. Second-strand cDNA synthesis was subsequently performed using DNA polymerase I and RNase H. Remaining overhangs were converted into blunt ends via exonuclease/polymerase activities. After adenylation of 3′ ends of DNA fragments, NEBNext Adaptor with hairpin loop structure was ligated to prepare for hybridization. To select cDNA fragments of preferentially 370 to 420 bp in length, the library fragments were purified with AMPure XP system (Beverly, USA). Then, 3 μl of USER Enzyme (NEB, USA) was used with size-selected, adaptor-ligated cDNA at 37°C for 15 min followed by 5 min at 95°C before PCR. Then, PCR was performed with Phusion High-Fidelity DNA polymerase, Universal PCR primers, and Index (X) Primer. At last, PCR products were purified (AMPure XP system), and library quality was assessed on the Agilent 5400 system (Agilent, USA) and quantified by qPCR (1.5 nM). The qualified libraries were pooled and sequenced on Illumina platforms with PE150 strategy, according to effective library concentration and data amount required. The experiment was conducted with ≥3 biological replicates.

### Total RNA isolation

Sorted cells were lysed in TRIzol reagent, followed by the addition of chloroform. After centrifugation at 4°C, the solution separated into distinct phases: RNA remained primarily in the upper aqueous phase, while DNA and proteins were retained in the interphase and lower organic phase. The colorless upper aqueous phase containing RNA was carefully transferred to a new tube, and RNA was precipitated with isopropyl alcohol. The RNA pellet was then washed with 75% ethanol to remove residual salts and organic solvents. After the ethanol was removed by centrifugation and air-dried, the RNA pellet was resuspended in RNase-free water. Samples were aliquoted and either immediately used for reverse transcription or stored at −80°C.

### RT-PCR and RT-qPCR

cDNA synthesis was performed using the HiScript III RT SuperMix for qPCR (+gDNA wiper) kit (Vazyme, no. R323) according to the manufacturer’s instructions. To eliminate genomic DNA contamination, 4× genomic DNA (gDNA) wiper mix was added to the initial reaction mixture. A 20-μl reverse transcription reaction was prepared by adding 5× HiScript III qRT SuperMix, followed by incubation at conditions specified by the manufacturer. The resulting cDNA was either used directly in qPCR or stored at −80°C.

PCR was conducted with TransStart FastPfu DNA Polymerase (TransGen, no. AP221), using a 10-fold dilution of the cDNA template. Reaction components and cycling conditions followed the supplier’s protocol. Primer details are provided in table S7.

For qPCR, ChamQ Universal SYBR qPCR Master Mix (no. Q711, Vazyme) was used, with reaction preparation per the manufacturer’s guidelines. A 20-μl reaction mixture was prepared, containing 10 μl of 2× ChamQ SYBR Master Mix, 0.5 μl each of 10 μM forward and reverse primers, 1 μl of diluted cDNA, and 8 μl of nuclease-free water. Cycling conditions were as follows: an initial denaturation at 95°C for 30 s, followed by 40 cycles of 95°C for 10 s and 60°C for 30 s. A melting curve analysis was included at the end of each qPCR run to confirm amplification specificity. Full primer usage details are available in table S8.

The experiment was conducted with ≥3 biological replicates.

### Protein-RNA complex prediction

AlphaFold3 server (https://alphafoldserver.com/) was used for structure prediction of SF3b-U2 snRNA-intron complex. For prediction, we submitted the SF3b complex (including SF3B1, SF3B2, SF3B3, SF3B4, SF3B5, SF3B6 and PHF5A), U2 snRNA, and a short segment of the intron ranging from the canonical 3′ss to ~100 bp upstream. The protein sequences were from UniProt. The random seed was set to its default state. The experiment was conducted with ≥3 biological replicates.

### Minigene assay

The minigene was constructed by inserting a DNA fragment containing the *NF1* genomic sequence from exon 34 to exon 35 into the pLVX-IRES-ZsGreen vector with mCherry. Mutagenesis of the minigene was performed using the Mut Express II Fast Mutagenesis Kit V2 (Vazyme, no. C214). HEK293T cells were initially transiently transfected with either *SF3B1* WT, R625H, or K700E variants. After 24 hours, GFP-positive cells were sorted, followed by a second transient transfection with the minigene in the sorted cells. mCherry-positive cells were then sorted 24 hours posttransfection.

Total RNA was isolated and reverse transcribed as previously described, followed by RT-PCR. Polyacrylamide gel electrophoresis (PAGE) was used for DNA fragment separation (~100 bp). The 20% polyacrylamide gel was prepared by combining 13.3 ml of 30% Acryl/Bis solution (Sangon Biotech, no. B546017), 4 ml of 5× Tris-borate-EDTA (TBE) buffer (Sangon Biotech, no. B548102), 2.5 ml of H2O, 140 μl of 10% ammonium persulfate (Sangon Biotech, no. A100486), and 13 μl of tetramethylethylenediamine (Sangon Biotech, no. A610508). Electrophoresis was conducted at a constant 200 V for ~150 min. The experiment was conducted ≥3 biological replicates.

### Western blot

Cells were seeded 48 hours before lysis and treated with 2% SDS (Sangon Biotech, no. B548118). Protein extracts were prepared by mixing with 6× SDS-PAGE Sample Loading Buffer (Beyotime, no. P0015F) and heating at 100°C for 10 min. Gels were cast using the TGX Stain-Free FastCast Acrylamide Kit, 10% (Bio-Rad, no. 1610183) (*44*). Proteins were transferred using 1× transfer buffer prepared with a 1:1:3 dilution of water, absolute ethanol, and Trans-Blot Turbo 5× Transfer Buffer (Bio-Rad, no. 10026938) onto 0.22-μm Immun-Blot PVDF Membranes (Bio-Rad, no. 1620177). Tris-Buffered Saline with Tween-20 (TBST) was prepared by adding 0.1% Tween 20 to tris-buffered saline. Membranes were blocked for 1 hour in a 3% bovine serum albumin (BSA) solution (3 g of BSA in 100 ml of TBST buffer).

Following blocking, membranes were incubated overnight at 4°C with primary antibodies diluted in 3% BSA buffer. After three 10-min washes with TBST, membranes were incubated with horseradish peroxidase–labeled goat anti-rabbit immunoglobulin G (H+L) (Beyotime, no. A0208) at 1:1000 dilution for 1 hour at room temperature. Protein visualization was performed using Clarity Western ECL Substrate (Bio-Rad, no. 1705060). Antibodies used for Western blotting are as follows: ERK (Cell Signaling Technology, no. 4695), phosphorylated ERK (Cell Signaling Technology, no. 4370), HSP90 (Cell Signaling Technology, no. 4877S), *SF3B1* (Abcam, no. ab202926), RASA1 (HUABIO, no. HA720052), and NF1 (Cell Signaling Technology, no. 14623).

### RAS activation assay

The Ras activation assay was performed using the Ras Activation Assay Kit (nonradioactive) (Merck Millipore, no. 17-218) following the manufacturer’s instructions. Briefly, 1 × 10^6^ cells were cultured per 15-cm dish and lysed with 0.5 to 1 ml of MLB (Mg2+ Lysis/Wash Buffer), ensuring nuclear lysis through repeated pipetting. The lysates were then collected and clarified by centrifugation at 14,000*g* for 5 min at 4°C. For the Ras pull-down assay, 5 to 10 μl of Ras Assay Reagent (RAF-1 RBD, agarose) was added to each lysate, followed by incubation at 4°C for 45 min. After incubation, the mixture was centrifuged at 14,000*g* for 10 s at 4°C, and the supernatant was discarded. The beads were washed three times with MLB and resuspended in 40 μl of 2× Laemmli buffer. To reduce disulfide bonds, 2 μl of 1 M dithiothreitol was added, and the samples were boiled for 5 min. The supernatant (20 μl) was then loaded onto a polyacrylamide gel for SDS-PAGE, and active Ras proteins were detected by Western blotting. All steps were performed on ice to prevent protein degradation.

### Immunohistochemistry (IHC) and H&E

Tumor tissues from patient-derived xenografts were fixed in 4% paraformaldehyde fixative (Sangon Biotech, no. E672002) and sectioned to a thickness of 5 μm using a Leica RM2235 Manual Rotary Microtome for routine sectioning. Immunohistochemistry (IHC) was performed using an Immunohistochemistry Kit (Sangon Biotech, no. D601037-0020), suitable for use with either rabbit or mouse primary antibodies (*45*). Hematoxylin and eosin (H&E) staining was conducted using the HE Staining Kit (Sangon Biotech, no. E60718). The antibodies used in IHC are as follows: Ki67 (HUABIO, no. HA721115) and ERK (Cell Signaling Technology, no. 4695). The experiment was conducted with over three biological replicates.

### Colony formation assay

Three thousand MUM2B cells overexpressing *SF3B1* WT, R625H, and K700E or control cells were seeded into each well of a six-well plate. The cells were incubated at 37°C in a humidified incubator with 5% CO_2_ until visible colonies formed, typically within 1 week. After colony formation, the cells were fixed with 4% paraformaldehyde fixative (Sangon Biotech, no. E672002) and stained with 0.1% crystal violet for 30 min to visualize the colonies. The experiment was conducted with over three biological replicates.

### Mice xenograft experiment

Six-week-old male nonobese diabetic mice (NOD)–severe combined immunodeficient (SCID) mice were purchased from Shanghai Jihui Laboratory Animals. Mice were randomly allocated into two groups with ≥6 animals per group to meet the minimum sample size requirements for *t* tests. MUM2B cells overexpressing *SF3B1* WT, R625H, and K700E were digested and suspended at a concentration of 1 × 10^7^ cells/ml. The cell suspensions were mixed with matrix gel (Absin, no. abs9495) in a 1:1 ratio and subcutaneously implanted into 6-week-old male NOD-SCID mice to generate the xenograft model. Tumor size was measured every 2 days, and the health conditions of the animals were closely monitored. All mice were housed in a pathogen-free environment and provided with ad libitum access to food and water. The subcutaneous inoculation of mice and tumor weighing were performed in a single-blind manner, while tumor photographing was an open-label operation. Mice with successful tumor engraftment and measurable tumor growth were included in the analysis. Animals that failed to develop tumors were excluded from the study. The animal care and experimental protocols were approved by the Ethics Committee of Shanghai Ninth People’s Hospital, affiliated with Shanghai Jiao Tong University, School of Medicine (reference no. SH9H-2022-A709-SB).

### Pan-cancer *SF3B1*–mutated sample collection

RNA-seq of patient samples with or without *SF3B1* hotspot mutations were collected from public portal resources including TCGA, ICGC (International Cancer Genome Consortium), and GEO (Gene Expression Omnibus). Detailed sample information is shown in table S1. For TCGA RNA-seq data, we downloaded aligned bam files from cancer genomics cloud (cgc.sbgenomics.com). This cohort includes 13 mutated (5 R625C and 8 R625H) UVM and five SKCM (two R625C and three R625H). In the meantime, equal number of WT samples was randomly chosen with the closest name ID of TCGA barcode for each mutated sample. Controlled accesses to TCGA raw mRNA sequencing data (phs000178.v11.p8) are approved with application project ID 35366. Data from eight patients with *SF3B1^K700E^* MDS and the same number of randomly chosen WT samples were obtained from GSE114922 (*46*). RNA-seq data of eight *SF3B1* K700E cases of patients with CLL were accessed from GSE92626 (*47*)/GSE116391 (*8*). Collectively, we have established transcriptomic characterization of Pan-cancer *SF3B1* mutations on 49 samples covered by four tumor types. Detailed sample information is provided in table S1.

### Identification of previously unidentified cryptic 3′ss usage

Cryptic 3′ss were computationally identified using a previously described method (*48*). This method enables highly sensitive detection of previously unidentified splice junctions and is therefore specifically designed for identification of cryptic 3′ss and 5′ss.

Briefly, we first downloaded the FASTQ files of the RNA-seq data (GEO accession GSE114922/GSE92626/GSE1163912) of patient samples with or without *SF3B1* mutation and then aligned the sequencing reads to the human genome (hg19) using STAR 2.7.4 (*49*), with a splice junction database (*7*). Counts of junction reads were obtained from the STAR output file (SJ.out.tab) and low-abundance junctions with a read coverage of <50 sum up across all samples were filtered out. For the remaining junctions, we defined it as a previously unidentified splice site if it only has one end (either 3′ or 5′) annotated in the provided known genome, while the other end is not. Corresponding canonical junction are found if it shares the same annotated end of the cryptic junction and the other end also annotated. If this kind of canonical junction did exist, we calculated the distance between the previously unidentified end and the corresponding canonical end. If there was more than one distance, we used the minimum. After adding the information of transcript’s strand and relative position to the canonical site, we determined whether a cryptic site is on 3′ss or 5′ss, upstream or downstream.

Next, the PSI values were calculated from the raw read counts. We then used *t* tests with PSI values (instead of raw read counts) to identify differential usage of cryptic 3′ss between *SF3B1*-mutated and WT samples, and the resulting *P* values were further adjusted by Benjamini-Hochberg (BH) multiple test correction to obtain *Q* values. Differences in mean PSI values were also calculated as important metrics for differential splicing. We used two threshold parameters (*P* < 0.05 and located closer than 50 nt upstream of the canonical 3′ss) to identify differentially used cryptic 3′ss upon *SF3B1* mutation. The identical analytical workflow was applied to RNA-seq data from NHM and K562 samples to identify cryptic 3′ss usage patterns associated with *SF3B1* mutations in these cell lines as well.

### Expression dataset and differential expression

To generate mRNA expression matrix for transcriptome analysis, we used featureCounts (*50*) from package Subread to call read counts from STAR realigned bam files. Genes with low read depths across the cohort were removed. Then, read counts were transformed into RPKM values followed by log_2_ transformation and quantile normalized on the sample level. Student’s *t* test was used to test for differential gene expression between sample groups, followed by BH multiple test correction. Differential expressed genes were considered to be significant if the adjusted *P* value is less than 0.05.

### Base preference calculation

To identify the sequence characteristics of *SF3B1^R625H/K700E^*-associated aberrant splicing targets, we used bedtools2.28 (*51*) to obtain the nucleotide sequences 100 bp upstream and 10 bp downstream of both the cryptic and associated canonical 3′ss. As a control, we selected 500 introns where no cryptic 3′ss usage was detected (*P* = 1 between *SF3B1* mutant and WT). For both the aberrant spliced targets and the control sequences, we calculated the nucleotide composition of adenine (A), thymine (T), guanine (G), and cytosine (C) at each position. We then used Fisher’s exact test to determine the significance of enrichment for each nucleotide in the aberrant group compared to the control group. Last, we visualized the nucleotide percentages at each position using the R package ggseqlogo.

### Functional enrichment analysis

Functional enrichment analyses including Gene Ontology, Kyoto Encyclopedia of Genes and Genomes, and Hallmark gene sets (Molecular Signatures Database, v6.1) were performed by the R package clusterProfiler. Terms with adjusted *P* < 0.01 were considered as significantly enriched.

### PCA and hierarchical clustering

To explore potential tumor/hotspot specificity or similarity on gene expression and 3′ss alteration, PCA and unsupervised hierarchical clustering were performed. PCA was applied to the expression or splicing matrix of NHM and K562 samples to inspect the gene expression difference or splicing difference between different *SF3B1* mutation types (WT/R625H/K700E) by using the prcomp function from the R package stats. For clustering analysis, we used the “Heatmap” command from the R package “ComplexHeatmap,” using Euclidean distance as the metric.

### Oncoprint maps

Mutation data for patients with CLL and UVM were downloaded from cBioPortal (www.cbioportal.org/). The oncoplot was generated using the “oncoPrint” command from R package ComplexHeatmap function in R. Statistical significance of co-occurrence was assessed using Fisher’s exact tests.

### Diversity, equity, ethics, and inclusion

All mice were housed in a pathogen-free environment and provided with ad libitum access to food and water. The animal care and experimental protocols were approved by the Ethics Committee of Shanghai Ninth People’s Hospital, affiliated with Shanghai Jiao Tong University, School of Medicine (reference no. SH9H-2022-A709-SB).
