# Supplementary material for: Spliceosomal mutation drives melanoma tumorigenesis via lineage-specific RAS activation
Source: Sci Adv. 2026 May 1;12(18):eadz8289. doi: 10.1126/sciadv.adz8289 (PMC13134632; doi:10.1126/sciadv.adz8289)
Supplement: Supplementary file 1 — Figs. S1 to S8 Legends for tables S1 to S8 [file sciadv.adz8289_sm.pdf]

Science Advances
AAAS

Supplementary Materials for

Spliceosomal mutation drives melanoma tumorigenesis via lineage-specific RAS activation

Ruixin Jiang *et al.*

Corresponding author: Hanlin Zeng, hanlin.zeng@shsmu.edu.cn; Zhaoqi Liu, liuzq@big.ac.cn; Peiqi Xing, xingpeiqi@big.ac.cn

Sci. Adv. **12**, eadz8289 (2026)
DOI: 10.1126/sciadv.adz8289

The PDF file includes:

Figs. S1 to S8
Legends for tables S1 to S8

Other Supplementary Material for this manuscript includes the following:

Tables S1 to S8

Fig. S1

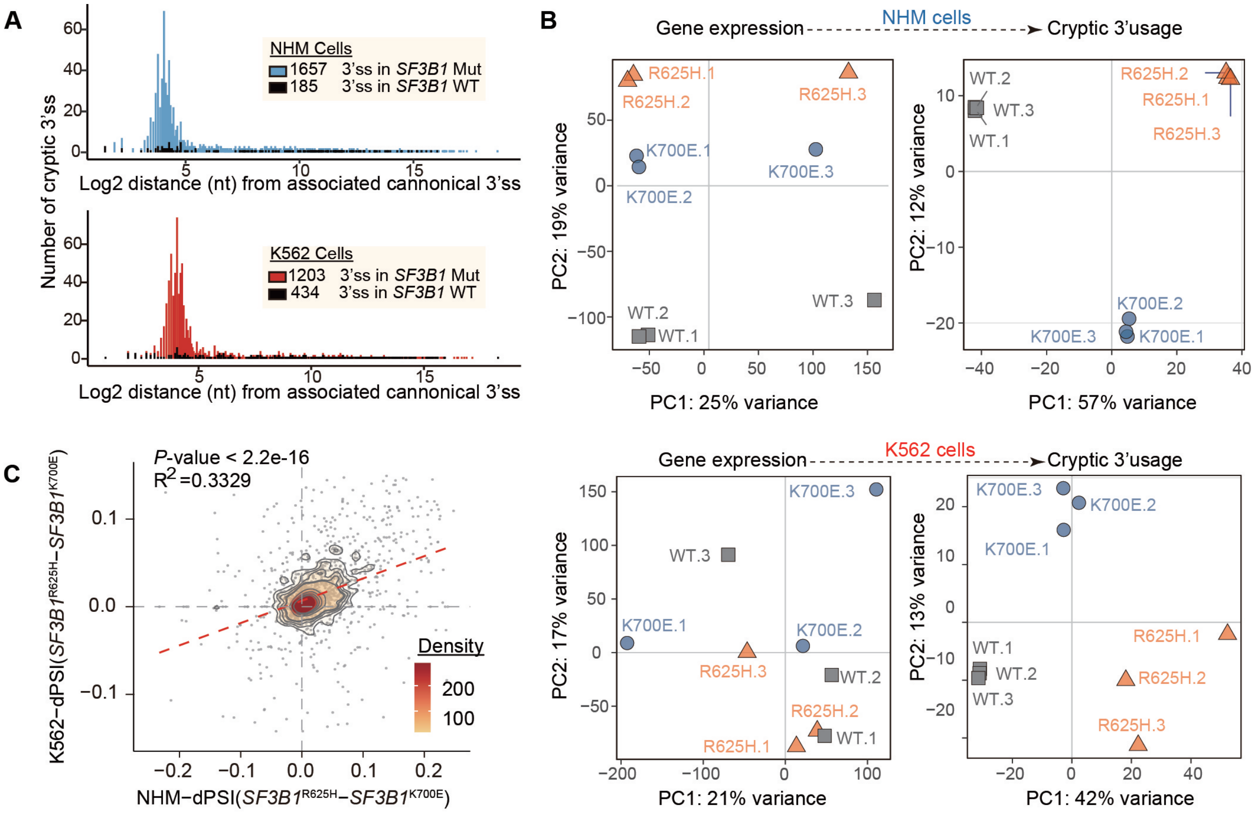
A
NHM Cells
1657 3'ss in SF3B1 Mut
185 3'ss in SF3B1 WT
K562 Cells
1203 3'ss in SF3B1 Mut
434 3'ss in SF3B1 WT
Number of cryptic 3'ss
Log2 distance (nt) from associated cannonical 3'ss
B
NHM cells
K562 cells
Gene expression
Cryptic 3'usage
PC1: 25% variance
PC2: 19% variance
PC1: 57% variance
PC2: 12% variance
PC1: 21% variance
PC2: 17% variance
PC1: 42% variance
PC2: 13% variance
R625H.1
R625H.2
R625H.3
K700E.1
K700E.2
K700E.3
WT.1
WT.2
WT.3
C
P-value < 2.2e-16
R2 =0.3329
Density
K562−dPSI(SF3B1R625H−SF3B1K700E)
NHM−dPSI(SF3B1R625H−SF3B1K700E)

Fig. S1. Systematic analysis of cryptic 3'ss usage and gene expression patterns associated with *SF3B1* mutations. (**A**) Density plot depicting the distribution of distances (in base pairs) between associated canonical 3'ss and cryptic 3'ss. (**B**) Principal component analysis (PCA) of gene expression (left) and Percent-Spliced-In (PSI) values for cryptic 3′ splice site (3′ss) events (right) across *SF3B1* mutation statuses in NHM and K562 cells. (**C**) Density scatter plot showing the correlation of differential PSI values (*SF3B1*^{R625H} -*SF3B1*^{K700E}) between NHM and K562 samples.

Fig. S2

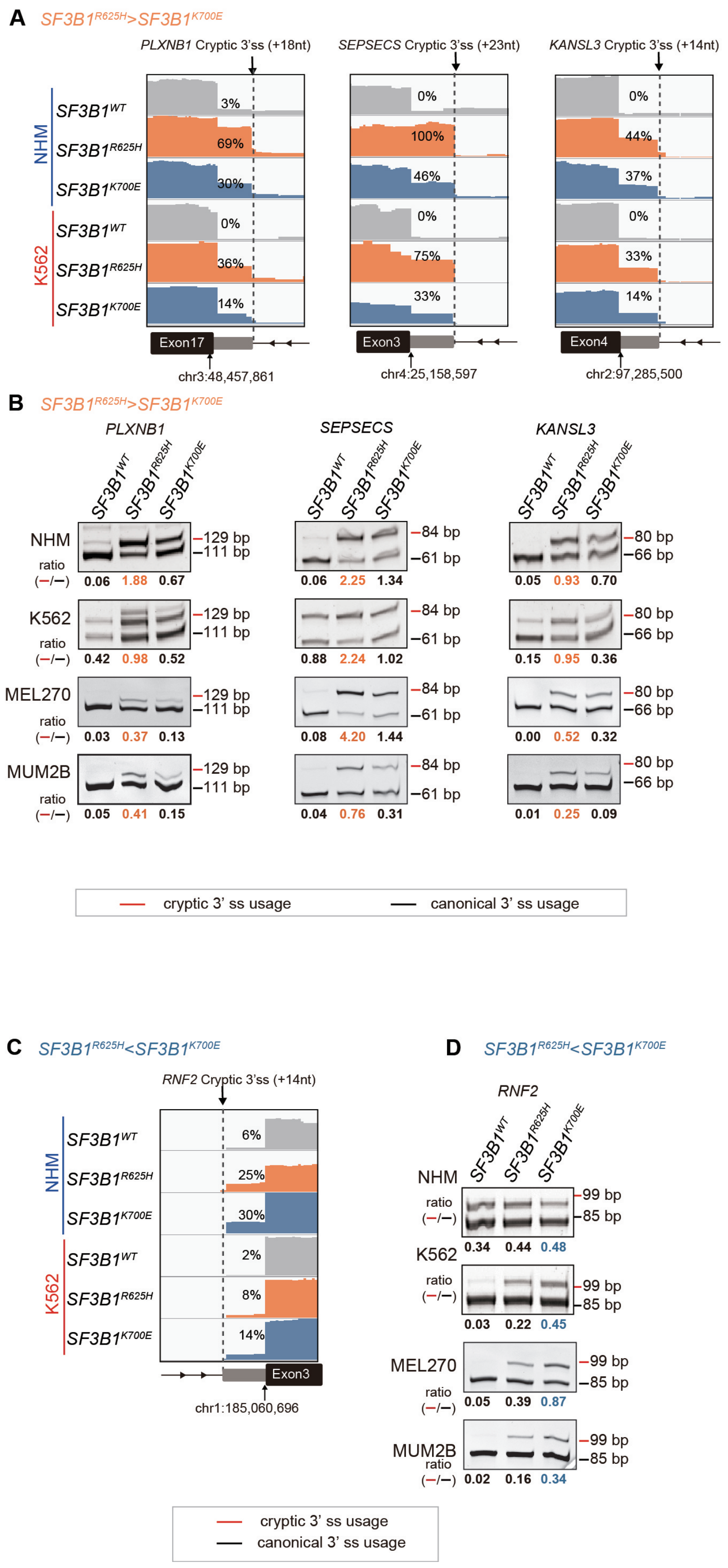
A SF3B1R625H>SF3B1K700E
PLXNB1 Cryptic 3'ss (+18nt)
SEPSECS Cryptic 3'ss (+23nt)
KANSL3 Cryptic 3'ss (+14nt)
NHM
K562
SF3B1WT
SF3B1R625H
SF3B1K700E
3%
69%
30%
0%
36%
14%
0%
100%
46%
0%
75%
33%
0%
44%
37%
0%
33%
14%
Exon17
Exon3
Exon4
chr3:48,457,861
chr4:25,158,597
chr2:97,285,500
B SF3B1R625H>SF3B1K700E
PLXNB1
SEPSECS
KANSL3
NHM
K562
MEL270
MUM2B
ratio
129 bp
111 bp
84 bp
61 bp
80 bp
66 bp
0.06 1.88 0.67
0.06 2.25 1.34
0.05 0.93 0.70
0.42 0.98 0.52
0.88 2.24 1.02
0.15 0.95 0.36
0.03 0.37 0.13
0.08 4.20 1.44
0.00 0.52 0.32
0.05 0.41 0.15
0.04 0.76 0.31
0.01 0.25 0.09
cryptic 3' ss usage
canonical 3' ss usage
C SF3B1R625H<SF3B1K700E
RNF2 Cryptic 3'ss (+14nt)
6%
25%
30%
2%
8%
14%
Exon3
chr1:185,060,696
D SF3B1R625H<SF3B1K700E
RNF2
99 bp
85 bp
0.34 0.44 0.48
0.03 0.22 0.45
0.05 0.39 0.87
0.02 0.16 0.34
cryptic 3' ss usage
canonical 3' ss usage

Fig. S2. Validation of splicing preference of between *SF3B1*$^{\mathbf{R625H}}$ and *SF3B1*$^{\mathbf{K700E}}$ mutation in NHM and K562 cells. (A-B), IGV plots (**A**) and RT-PCR (**B**) validation of representative *SF3B1*^{R625H} preferred cryptical 3'ss events in NHM and K562 cells. **c-d**, IGV plots (**C**) and RT-PCR (**D**) of representative *SF3B1*^{K700E} preferred cryptical 3'ss events in NHM and K562 cells.

Fig. S3

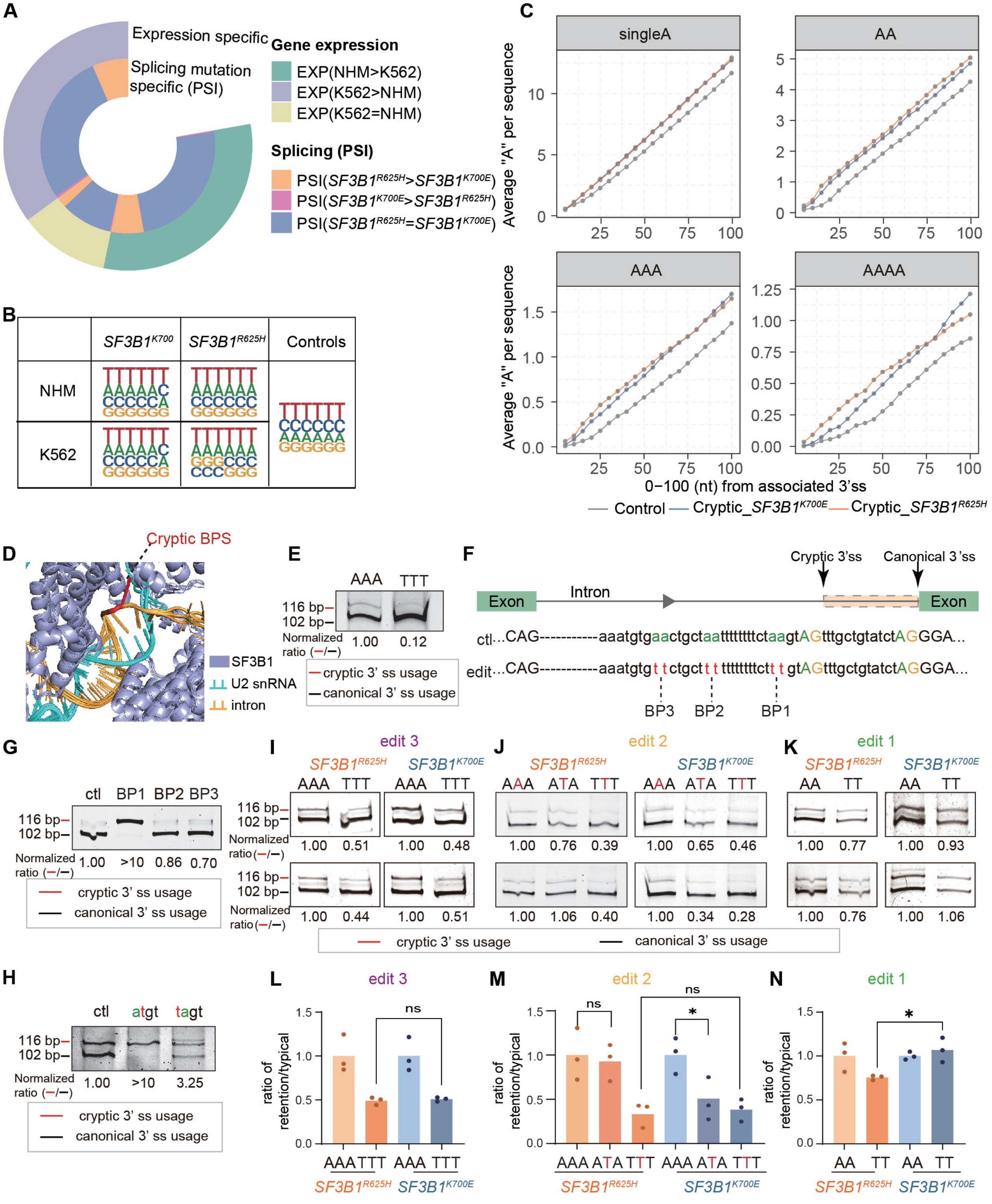

A
Expression specific
Splicing mutation specific (PSI)
Gene expression
EXP(NHM>K562)
EXP(K562>NHM)
EXP(K562=NHM)
Splicing (PSI)
PSI(SF3B1R625H>SF3B1K700E)
PSI(SF3B1K700E>SF3B1R625H)
PSI(SF3B1R625H=SF3B1K700E)
B
SF3B1K700
SF3B1R625H
Controls
NHM
K562
C
singleA
AA
AAA
AAAA
Average "A" per sequence
0–100 (nt) from associated 3'ss
Control
Cryptic_SF3B1K700E
Cryptic_SF3B1R625H
D
Cryptic BPS
SF3B1
U2 snRNA
intron
E
AAA
TTT
116 bp
102 bp
Normalized ratio (—/—)
1.00
0.12
cryptic 3' ss usage
canonical 3' ss usage
F
Cryptic 3'ss
Canonical 3'ss
Exon
Intron
Exon
ctl...CAG-----------aaatgtgaactgctaattttttttctaagtAGtttgctgtatctAGGGA...
edit...CAG-----------aaatgtgttctgcttttttttttcttgtAGtttgctgtatctAGGGA...
BP3
BP2
BP1
G
ctl
BP1
BP2
BP3
116 bp
102 bp
Normalized ratio (—/—)
1.00
>10
0.86
0.70
cryptic 3' ss usage
canonical 3' ss usage
H
ctl
atgt
tagt
116 bp
102 bp
Normalized ratio (—/—)
1.00
>10
3.25
cryptic 3' ss usage
canonical 3' ss usage
I
edit 3
SF3B1R625H
SF3B1K700E
AAA
TTT
AAA
TTT
1.00
0.51
1.00
0.48
1.00
0.44
1.00
0.51
J
edit 2
SF3B1R625H
SF3B1K700E
AAA
ATA
TTT
AAA
ATA
TTT
1.00
0.76
0.39
1.00
0.65
0.46
1.00
1.06
0.40
1.00
0.34
0.28
K
edit 1
SF3B1R625H
SF3B1K700E
AA
TT
AA
TT
1.00
0.77
1.00
0.93
1.00
0.76
1.00
1.06
cryptic 3' ss usage
canonical 3' ss usage
L
edit 3
ratio of retention/typical
ns
AAA TTT AAA TTT
SF3B1R625H
SF3B1K700E
M
edit 2
ns
ns
*
AAA ATA TTT AAA ATA TTT
SF3B1R625H
SF3B1K700E
N
edit 1
*
AA TT AA TT
SF3B1R625H
SF3B1K700E

Fig. S3. Poly-adenine enrichment upstream of cryptic AG sites enhances cryptic 3′ splice site (3′ss) usage by *SF3B1*$^{\mathbf{R625H}}$. (**A**) The PSI values of cryptic 3’ss events and the expression levels of their corresponding genes exhibit distinct distributions across different cell types and mutation types. Outer circle: Genes associated with cryptic 3′ss events are categorized into three groups based on expression preferences (NHM-preferential, K562-preferential, or equally expressed). Inner circle: Cryptic 3′ss events corresponding to these genes are further grouped based on PSI preferences (higher PSI under *SF3B1*^{R625H}, higher PSI under *SF3B1*^{K700E}, or equal PSI). (**B**) Contingency table showing the enrichment of 6-mer motif located 30 bp upstream of the cryptic AG site in cryptic 3′ss events associated with different *SF3B1* mutations in isogenic NHM or K562 cells. (**C**) Average frequencies of motif features (“A”, “AA”, “AAA”, “AAAA”) within 100 bp upstream of cryptic AG site preferentially used by *SF3B1*^{K700E} or *SF3B1*^{R625H} mutations, as well as control AG sites without upstream aberrant 3’ss usage. (**D**) Aligned structure of five predicted results by Alphafold3. The alignment was conducted using the alignment tool in PyMOL. (**E**) RT-PCR validation of the cryptic branch point. (**F**) Schematic diagram of sequence editing to explore the canonical BPS. (**G**) AA to TT mutation of the three predicted BPS (BP1, BP2, and BP3) upstream of canonical 3’ss within the minigene followed by RT-PCR validation of splicing efficiency. (**H**) Individual A to T mutation of the BP1 sequences within the minigene followed by RT-PCR validation of splicing efficiency. (**I-N**) RT-PCR based DNA gel result (**I-K**) and quantification of splicing outcomes for minigenes (**L-N**) with specific edits under different *SF3B1* mutation contexts in isogenic 293T cells (n=3; ns, no significance; *P < 0. 05).

Fig. S4

A

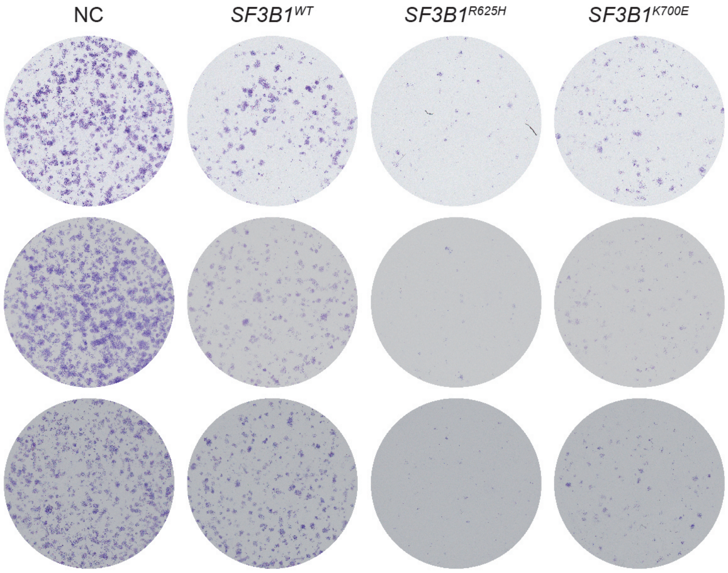

B

Chronic Lymphocytic Leukemia (IUOPA, Nature 2015)

Samples with mutation data (506 patients) - *SF3B1&ATM&TP53*

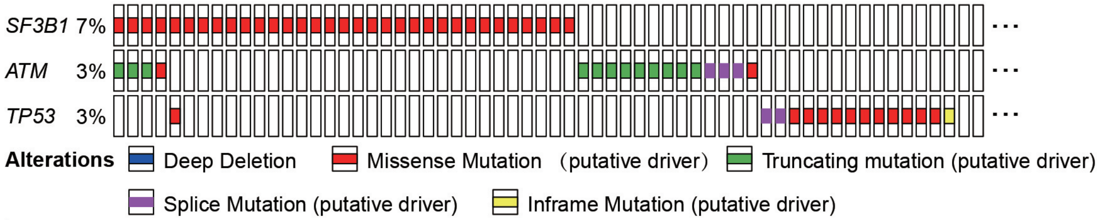

A	B	Neither	A Not B	B Not A	Both	Log2 Odds Ratio	p-Value	Tendency
SF3B1	*ATM*	460	29	13	4	2.29	0.019	Co-occurrence
SF3B1	*TP53*	459	32	14	1	0.034	1	Co-occurrence

C

Uveal Melanoma (TCGA, PanCancer Atlas)

Samples with mutation data (80 patients) - *SF3B1&ATM*

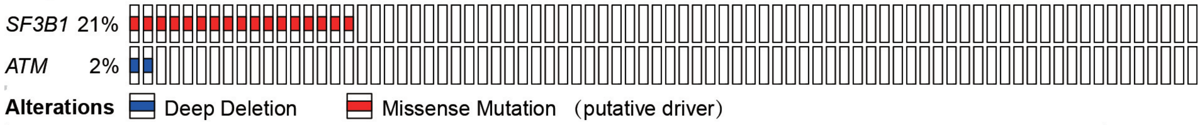

A	B	Neither	A Not B	B Not A	Both	Log2 Odds Ratio	p-Value	Tendency
SF3B1	*ATM*	61	17	0	2	>3	0.054	Co-occurrence

Fig. S4. Cells transduced with wild-type or mutant SF3B1 constructs only can hardly survive. (**A**) Colony formation assay comparing MUM2B cells transduced with empty vector or SF3B1 constructs carrying the indicated mutations. (**B-C**) Oncoplot showing mutations or deep deletions in the putative driver genes *SF3B1*, *ATM*, and *TP53* in cohorts of chronic lymphocytic leukemia (IUOPA) (**B**) and uveal melanoma (TCGA) (**C**). The table below the oncoplot summarizes the co-occurrence probabilities of *SF3B1* with either *ATM* or *TP53* (Fisher's exact tests).

Fig. S5

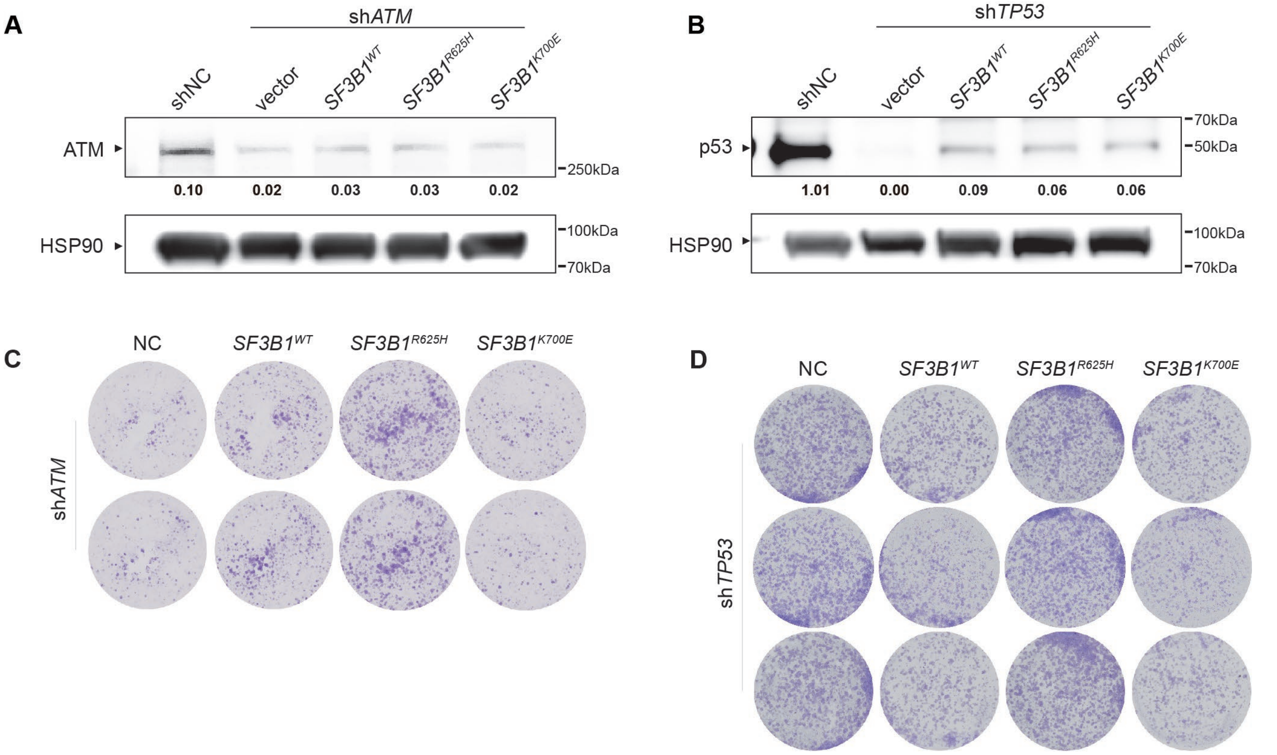
A
shATM
shNC
vector
SF3B1WT
SF3B1R625H
SF3B1K700E
ATM
250kDa
0.10
0.02
0.03
0.03
0.02
HSP90
100kDa
70kDa
B
shTP53
shNC
vector
SF3B1WT
SF3B1R625H
SF3B1K700E
p53
70kDa
50kDa
1.01
0.00
0.09
0.06
0.06
HSP90
100kDa
70kDa
C
NC
SF3B1WT
SF3B1R625H
SF3B1K700E
shATM
D
NC
SF3B1WT
SF3B1R625H
SF3B1K700E
shTP53

Fig. S5. Comparison colony formation under different *SF3B1*^{R625H} mutation status in MUM2B cells. (**A-B**) Western blot validation of shRNA-mediated knockdown efficiency for *ATM* (**A**) and TP53 (**B**). Quantitative analyses normalized to HSP90 are labeled below related band. (**C-D**) Representative images from triplicate colony formation assays comparing MUM2B cells transduced with empty vector or SF3B1 constructs carrying the indicated mutations in the context of *ATM* (**C**) or *TP53* (**D**) knockdown.

Fig. S6

A

Uveal Melanoma (TCGA, PanCancer Atlas)

Samples with mutation data (80 patients) - SF3B1&GNAQ&GNA11

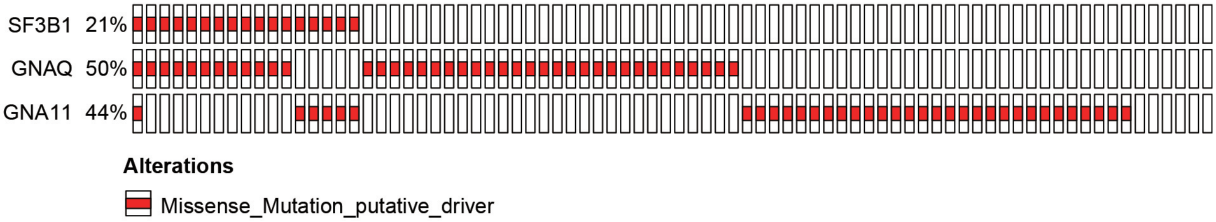

A	B	Neither	A Not B	B Not A	Both	Log2 Odds Ratio	p-Value	Tendency
SF3B1	GNAQ	35	5	28	12	1.59	0.099	Co-occurrence
SF3B1	GNA11	34	11	29	6	-0.645	0.583	Mutual Exclusivity
GNAQ	GNA11	6	39	34	1	-7.788	0.000	Mutual Exclusivity

B

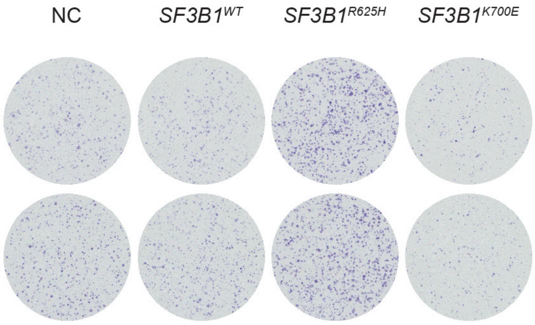

Fig. S6. Comparison colony formation under different *SF3B1* mutation status in GNAQ mutated cells. (**A**) Oncoplot showing mutations in the putative driver genes *SF3B1*, *GNAQ*, and *GNA11* in cohorts of uveal melanoma (TCGA). The table below the oncoplot summarizes the co-occurrence probabilities of *SF3B1* with either *ATM* or *TP53*. (Fisher's exact tests). (**B**) Representative images from triplicate colony formation assays comparing MEL270 cells (with *GNAQ* mutation) transduced with empty vector or SF3B1 constructs carrying the indicated mutations.

Fig. S7

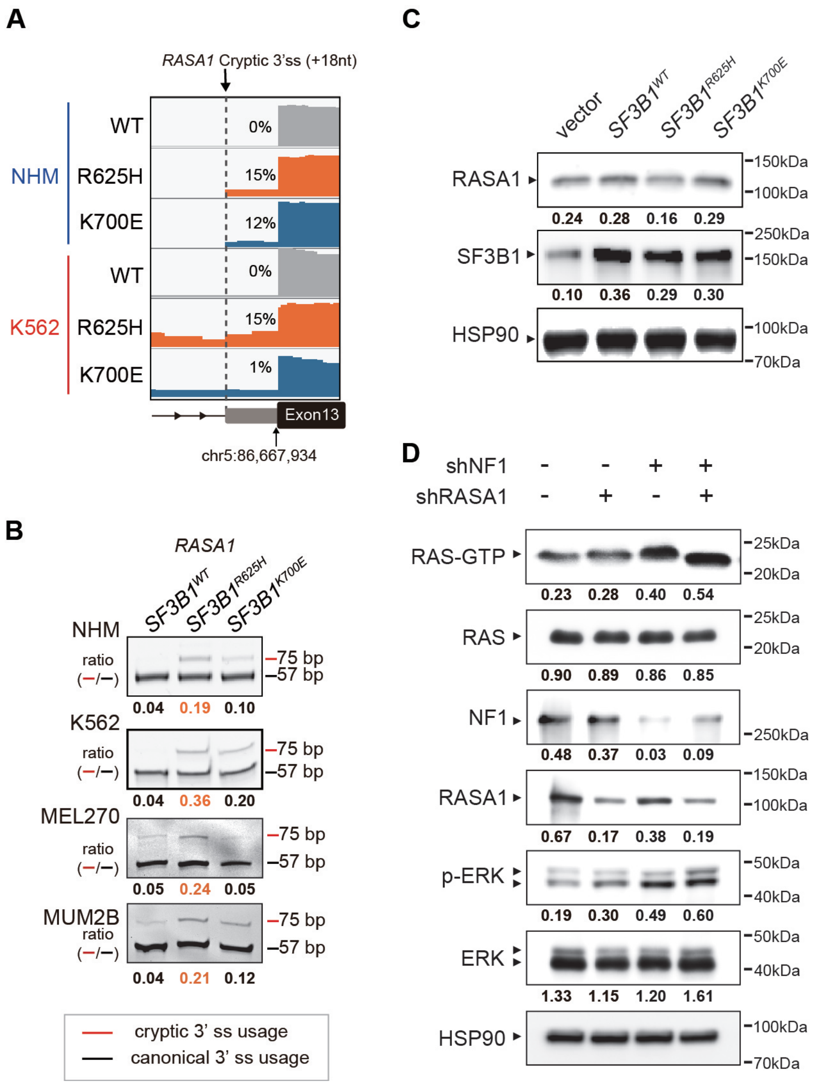
A
RASA1 Cryptic 3'ss (+18nt)
NHM
WT
0%
R625H
15%
K700E
12%
K562
WT
0%
R625H
15%
K700E
1%
Exon13
chr5:86,667,934
B
RASA1
SF3B1WT
SF3B1R625H
SF3B1K700E
NHM
ratio
(—/—)
75 bp
57 bp
0.04 0.19 0.10
K562
ratio
(—/—)
75 bp
57 bp
0.04 0.36 0.20
MEL270
ratio
(—/—)
75 bp
57 bp
0.05 0.24 0.05
MUM2B
ratio
(—/—)
75 bp
57 bp
0.04 0.21 0.12
cryptic 3' ss usage
canonical 3' ss usage
C
vector
SF3B1WT
SF3B1R625H
SF3B1K700E
RASA1
150kDa
100kDa
0.24 0.28 0.16 0.29
SF3B1
250kDa
150kDa
0.10 0.36 0.29 0.30
HSP90
100kDa
70kDa
D
shNF1 - - + +
shRASA1 - + - +
RAS-GTP
25kDa
20kDa
0.23 0.28 0.40 0.54
RAS
25kDa
20kDa
0.90 0.89 0.86 0.85
NF1
250kDa
0.48 0.37 0.03 0.09
RASA1
150kDa
100kDa
0.67 0.17 0.38 0.19
p-ERK
50kDa
40kDa
0.19 0.30 0.49 0.60
ERK
50kDa
40kDa
1.33 1.15 1.20 1.61
HSP90
100kDa
70kDa

Fig. S7. *SF3B1*^{R625H} induces stronger mis-splicing of *RASA1* than *SF3B1*^{K700E}, which cooperates with *NF1* mis-splicing to mediates RAS activation. (**A**) IGV plots showing cryptic 3'ss events on *RASA1* induced by *SF3B1*^{K700E} or *SF3B1*^{R625H} mutations in isogenic NHM and K562 cells. (**B**) RT-PCR validation of cryptic 3'ss events of *RASA1* in isogenic NHM and K562 cells transfected with different *SF3B1* variant. (**C**)Western blot detection of *RASA1* down-regulation induced by WT or mutant *SF3B1*. Quantitative analyses normalized to HSP90 are labeled below related band. (**D**) RAS activation assay in MUM2B cells with *NF1* and/or *RASA1* knockdown.

Fig. S8

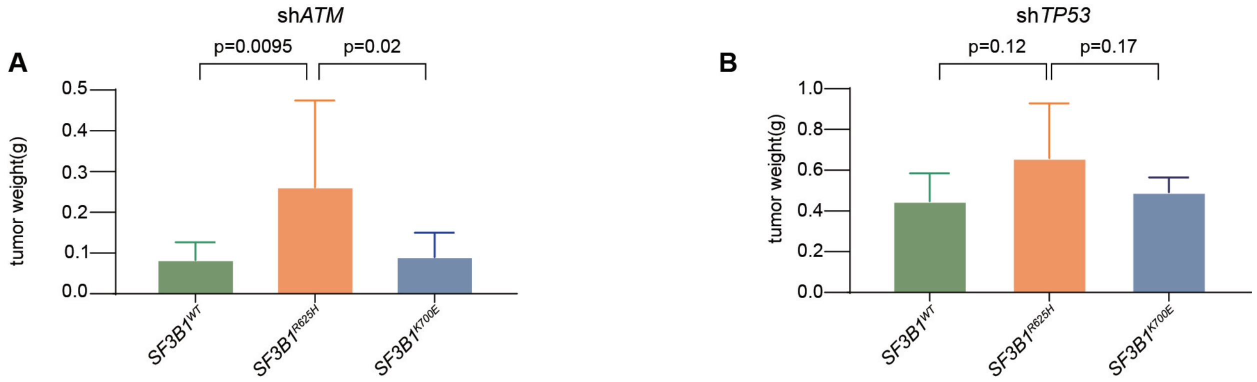

A
shATM
p=0.0095
p=0.02
tumor weight(g)
0.5
0.4
0.3
0.2
0.1
0.0
SF3B1WT
SF3B1R625H
SF3B1K700E
B
shTP53
p=0.12
p=0.17
tumor weight(g)
1.0
0.8
0.6
0.4
0.2
0.0
SF3B1WT
SF3B1R625H
SF3B1K700E

Fig. S8. *SF3B1*^{R625H} mutation promotes melanoma progression in MUM2B CDX model. (A) Tumor weights of MUM2B cell line-derived xenografts (CDXs) expressing *SF3B1* constructs with the indicated mutations in the context of *ATM* knockdown. (B) Tumor weights of MUM2B CDXs expressing *SF3B1* constructs with the indicated mutations in the context of *TP53* knockdown.

Supplementary Tables

Table S1. Information of SF3B1 mutated pan-cancer samples and cell lines.

Table S2. Cryptic 3' splice sites in pan-cancer SF3B1 mutated samples and corresponding PSI matrix.

Table S3. Cryptic 3' Splice Sites in SF3B1 mutated NHM and K562 samples and corresponding PSI matrix.

Table S4. Genes expression matrix in SF3B1 WT and mutant NHM and K562 samples.

Table S5. Differentially expressed genes between SF3B1 mutant vs WT UVM and SKCM from the TCGA cohort.

Table S6. Differentially expressed genes between SF3B1 mutant vs WT NHM and K562 cell-lines.

Table S7. Primers for RT-PCR.

Table S8. Primers for RT-qPCR.
